# Dexmedetomidine Rapidly Relieves Stress‐Induced Hyperalgesia via Presynaptic *α*2‐Adrenergic Inhibition at Orbitofrontal–Insular Glutamatergic Synapses

**DOI:** 10.1002/mco2.70848

**Published:** 2026-06-30

**Authors:** Hui Rong, Yang‐Xun Zhang, Huijie Zhu, Yinyao Li, Yunfan Hou, Yun‐Yong Xie, Lu‐Yao Li, Bei‐Bei Zhang, Shu‐Tao Xie, Wei Zhang, Qi‐Peng Zhang, Xiao‐Yang Zhang, Jing‐Ning Zhu, Xiaoping Gu, Zhengliang Ma

**Affiliations:** ^1^ Department of Anesthesiology, Nanjing Drum Tower Hospital Affiliated Hospital of Medical School, Nanjing University Nanjing China; ^2^ State Key Laboratory of Pharmaceutical Biotechnology, National Resource Center for Mutant Mice, Department of Anesthesiology, Nanjing Drum Tower Hospital, Institute for Brain Sciences, and Department of Physiology, School of Life Sciences Nanjing University Nanjing China

**Keywords:** anterior insula cortex, dexmedetomidine, hyperalgesia, orbitofrontal cortex, plasticity, stress

## Abstract

Dexmedetomidine (Dex), an *α*2‐adrenergic receptor (*α*2‐AR) agonist, is widely used for its antihyperalgesic effects in perioperative pain management, yet its underlying mechanisms remain largely undefined. Here, we identify a rapid, circuit‐specific mechanism by which Dex reverses stress‐induced hyperalgesia (SIH) in mice. Stress exposure strengthens excitatory drive from the orbitofrontal cortex (OFC) to the anterior insula cortex (AIC), shifting the excitatory/inhibitory balance toward excitation and increasing the intrinsic excitability of AIC glutamatergic neurons. Viral tracing and optogenetics reveal a direct OFC–AIC glutamatergic projection. Optogenetic or chemogenetic activation of this pathway in otherwise naïve mice potentiates glutamatergic synaptic transmission and is sufficient to induce hyperalgesia, phenotyping SIH. Dex rapidly suppresses these effects by engaging presynaptic *α*2‐ARs on OFC terminals, thereby reducing transmitter release at OFC–AIC synapses. Consistently, chemogenetic silencing of AIC neurons or of the OFC–AIC projection alleviates hyperalgesia in SIH mice. These findings define the OFC–AIC glutamatergic circuit as a key substrate for stress‐related pain amplification and uncover a rapid presynaptic *α*2‐AR “brake” as the mechanism underlying Dex's antihyperalgesic action, highlighting a tractable therapeutic entry point for stress‐exacerbated pain states.

## Graphical abstract

This study identified a maladaptive OFC–AIC glutamatergic circuit in stress‐induced hyperalgesia induction. This critical circuit provides an α2‐AR‐dependent braking target for dexmedetomidine to alleviate excessive glutamatergic transmission and hyperalgesia.

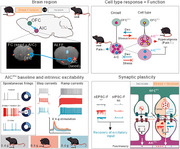

## 1 Introduction

Hyperalgesia, defined by the International Association for the Study of Pain as an exaggerated response to noxious stimuli, is a common yet often underrecognized complication in surgical patients, affecting up to 234 million people worldwide [1]. In perioperative settings, hyperalgesia contributes to a cascade of adverse outcomes, including nausea, ileus, pulmonary complications, delayed ambulation, prolonged hospitalization, impaired rehabilitation, and increased risk for chronic postsurgical pain syndromes [2]. Although psychological stressors such as preoperative anxiety and depression are known clinical risk factors [3], the underlying neurobiological mechanisms of stress‐induced hyperalgesia (SIH) remain poorly understood, impeding the development of effective interventions.

The anterior insula cortex (AIC) has emerged as a critical brain region for integrating the emotional and sensory aspects of pain [4]. Human neuroimaging studies have linked AIC activity to mood fluctuations in healthy individuals and enhanced responses to uncontrollable pain [5], while studies on patients with ischemic lesions in the AIC show blunted emotional responses and reduced pain withdrawal behaviors [6]. The cytoarchitecture of the AIC mainly consists of excitatory CaMKII*α* ^+^ glutamatergic pyramidal neurons and diverse types of inhibitory interneurons (e.g., SOM^+^, PV^+^, or VIP^+^). These pyramidal neurons integrate long‐range excitatory inputs with local inhibitory signals to shape adaptive pain responses and are implicated in encoding empathic and affective pain states [7, 8, 9]. However, it remains unclear how preoperative psychological stressors, such as anxiety or fear of the outcomes of the operation, anesthesia failure, or the unknown [10, 11], disrupt the excitatory/inhibitory balance within AIC and contribute to the pathogenesis of SIH.

Dexmedetomidine (Dex), a selective *α*2‐adrenergic receptor (*α*2‐AR) agonist, is widely used in anesthesia and critical care for its sedative and analgesic properties without causing respiratory depression [12, 13, 14]. Unlike opioids, which can paradoxically exacerbate hyperalgesia via maladaptive plasticity [15, 16], Dex reduces opioid consumption [17], suggesting a mechanistically distinct and potentially advantageous action. A deeper understanding of its neural mechanism is therefore essential for the development of targeted therapeutic strategies that can directly modulate its central sites of action. While the sedative effects of Dex have been linked to neuronal hyperpolarization in the locus coeruleus (LC) [18], the cortical cellular and circuit mechanisms underlying its antihyperalgesic properties remain largely unexplored. Therefore, in this study, we aim to elucidate how preoperative stress alters excitatory and inhibitory synaptic inputs and intrinsic excitability within AIC microcircuits, and how these changes contribute to the pathogenesis of postoperative hyperalgesia. We further investigate how Dex exerts its rapid antihyperalgesic effects by modulating activity within these stress‐responsive circuits. Our findings reveal critical insights into the neurobiological underpinnings of SIH and highlight novel therapeutic targets for fast‐acting, circuit‐based interventions in perioperative pain management.

While the sedative effects of Dex have been linked to neuronal hyperpolarization in the LC [18], its cortical cellular and circuit mechanisms underlying its antihyperalgesic properties remain largely unexplored. Here, we sought to determine how preoperative stress reshapes excitatory and inhibitory synaptic inputs, as well as intrinsic excitability, within AIC microcircuits, and how these changes contribute to the pathogenesis of postoperative hyperalgesia. We further investigate how Dex exerts its rapid antihyperalgesic effects by modulating activity within these stress‐responsive circuits. Our findings reveal critical insights into the neurobiological underpinnings of SIH and highlight novel therapeutic targets for fast‐acting, circuit‐based interventions in perioperative pain management.

## 2 Results

### 2.1 Systemic Administration of Dex Rapidly Relieves SIH

To establish an animal model for SIH, we employed a modified single prolonged stress (SPS) paradigm, comprising restraint, forced swim, anesthesia, and plantar shock, followed by a right (ipsilateral) plantar incision in mice. To validate the efficacy of SPS in inducing anxiety‐like behaviors, we performed the open field test (OFT) and the elevated plus maze (EPM) test 1 day poststress exposure. SPS‐treated mice exhibited significantly reduced time spent in the center zone of the OFT and the open arms of the EPM, along with fewer entries into these zones, compared with control (Ctrl) mice (Figure S1A–H). In addition, the total locomotor distance did not change in SPS mice (Figure S1D,H), indicating that the observed behavioral changes were due to anxiety rather than motor impairments.

**FIGURE 1:**
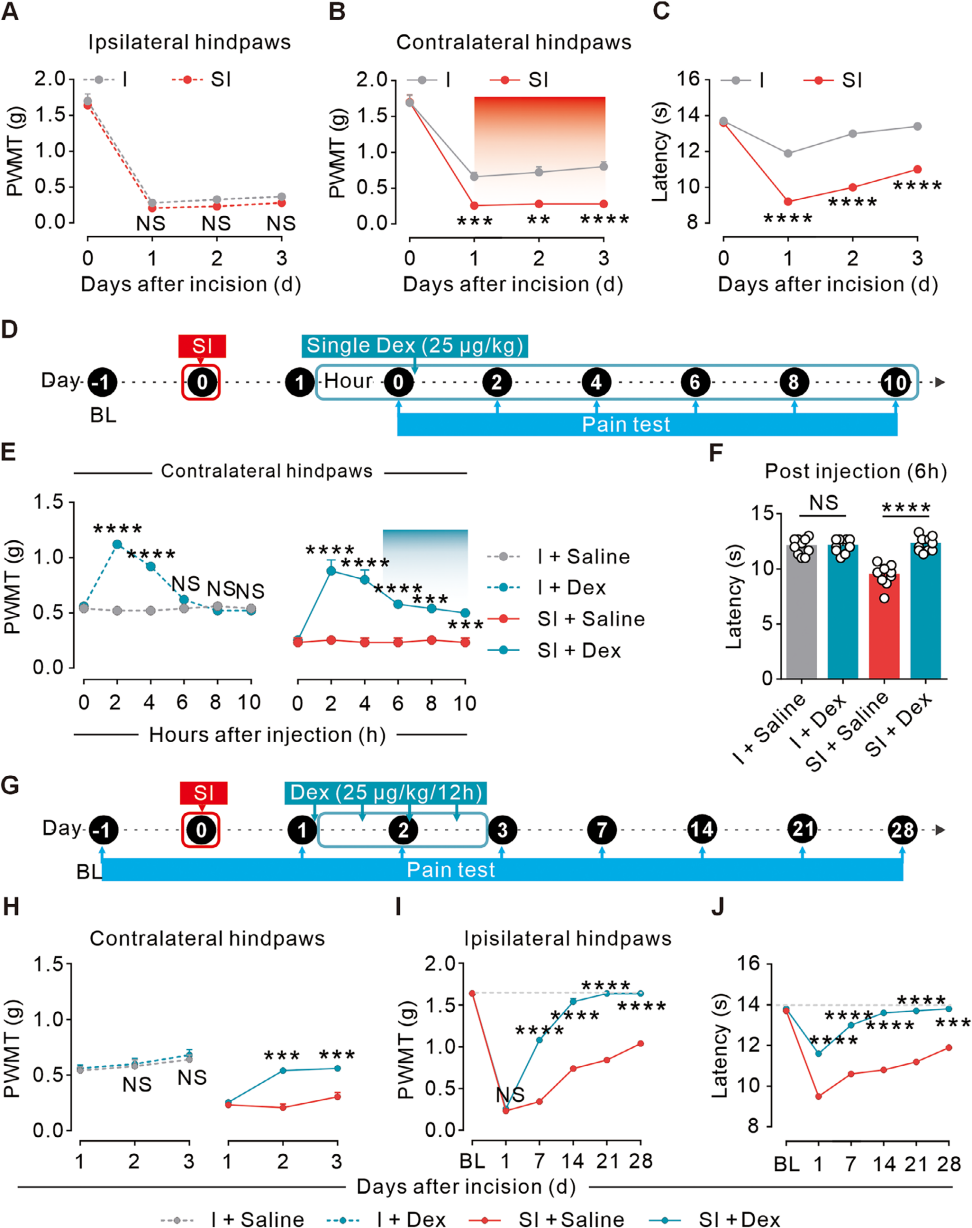
Systemic administration of Dex produces rapid and sustained analgesic effects in SIH mice. (A) Dynamic changes of paw withdrawal mechanical threshold (PWMT) in the ipsilateral in stress‐induced hyperalgesia (SIH) mice. *n* = 10 mice per group. Preoperative single prolonged stress (SPS) followed by a right (ipsilateral) plantar incision on the next day was used to induce SIH. SI, stress + incision. I, incision only. (B) Dynamic changes of PWMT in the contralateral hindpaws in SIH mice. *n* = 10 mice per group. (C) Dynamic changes of thermal withdrawal latency in SIH mice. *n* = 10 mice per group. (D) Experimental timeline showing single dexmedetomidine (Dex) administration and behavioral assessments in I or SI mice. (E) Contralateral PWMT after single saline or Dex administration to evaluate pure analgesic effects and antihyperalgesic effects on Day 1 after surgery in I or SI mice. *n* = 10 mice per group. (F) Thermal withdrawal latency measured at 6 h after single saline or Dex administration in I or SI mice. *n* = 10 mice per group. (G) Experimental timeline showing intermittent Dex administration in early days, and behavioral assessments in recovery days in I or SI mice. (H) Contralateral PWMT after intermittent saline or Dex administration to evaluate sustained antihyperalgesic effects in the early days in SI mice. *n* = 10 mice per group. (I) Ipsilateral PWMT after intermittent saline or Dex administration to evaluate sustained antihyperalgesic effects in 28 days in SI mice. *n* = 10 mice per group. (J) Thermal withdrawal latency measured after intermittent saline or Dex administration to evaluate sustained antihyperalgesic effects in 28 days in SI mice. *n* = 10 mice per group. Data are presented as mean ± SEM. NS, no significant. **p < 0.01, ***p < 0.001, ****p < 0.0001. Statistical analysis: two‐way repeated‐measures ANOVA followed by Bonferroni's posthoc test (A–C, E, and H, I) and two‐tailed unpaired *t*‐tests (F). Source data are provided in the Supporting Information.

We next examined the impact of preoperative SPS on postoperative pain sensitivity in hindpaws of mice that underwent a right plantar incision. In von Frey tests, no significant differences in paw withdrawal mechanical thresholds (PWMT) were detected at the ipsilateral (incised) hindpaw between the incision‐only (I) and SPS + incision (SI) groups during the early postoperative period (Days 1–3) (Figure 1A). This may be attributable to the limited sensitivity of von Frey hairs at extremely low gram force ranges. Remarkably, however, mice in the SI group exhibited a significantly lower mechanical pain threshold in the contralateral (nonincised) hindpaw compared with the I group (Figure 1B), indicating a generalized sensitization effect induced by SPS. Consistent with previous reports [19, 20], thermal pain sensitivity was also increased by stress exposure. Mice in the SI group displayed significantly shorter withdrawal latencies to noxious thermal stimuli than those in the I group, in both contralateral and ipsilateral hindpaws (Figure 1C). These results collectively support that preoperative SPS effectively induces anxiety‐like behaviors and enhances both localized and widespread postoperative pain sensitivity, modeling key features of SIH.

To directly assess SIH on the ipsilateral (incised) hindpaw, we developed a fentanyl‐based pharmacological test. This design was inspired by clinical observations that patients experiencing preoperative anxiety‐related hyperalgesia often require higher doses of postoperative opioid analgesics [15]. We hypothesized that an optimal dose of fentanyl could serve as a functional discriminator, selectively elevating PWMT in mice of the I group, but not in the SI group, thus revealing heightened pain sensitivity in the latter group. To test this, we administered graded doses of fentanyl (3.12–50 µg/kg, i.p.) to group I mice for the optimal dose, which was then tested in group SI mice. As expected, a dose of 12.5 µg/kg was sufficient to significantly increase the PWMT in group I mice, compared with saline controls (Figure S1I). However, this analgesic effect was completely absent in group SI mice (Figure S1J), indicating that the same dose of fentanyl was ineffective under SIH conditions. These findings provide compelling evidence that SPS reduces mechanical thresholds in both ipsilateral and contralateral paws postincision, consistent with a global enhancement of pain sensitivity. Given the difficulty of accurately measuring threshold changes at extremely low gram ranges in the incised hindpaw, and in light of the synchronized pain sensitization observed across both hindpaws, we determined that pain responses in the contralateral hindpaw provide a reliable and sensitive surrogate measure of postoperative hyperalgesia for subsequent behavioral analyses.

To assess the effect of systemic Dex on SIH, we administered Dex at a light sedative dose (25 µg/kg, i.p.) to SI mice, following a previously validated protocol [21]. We first evaluated the acute response to a single Dex injection on PWMT in both I and SI groups (Figure 1D,E). Within 4 h postinjection, Dex produced a robust antinociceptive effect in both groups, as reflected by increased PWMT. However, after 6 h, a divergence emerged: SI mice maintained elevated PWMT, whereas I mice returned to baseline levels (Figure 1E). To minimize the influence of acute analgesia, we selected the 6‑h time point to assess sustained effects on thermal sensitivity (Figure 1F). At this time point, hot‐plate tests revealed a persistent reduction in thermal hyperalgesia in SI mice, but no significant effect in the I group (Figure 1F). We next examined whether early postoperative intervention could influence pain progression, given that acute postsurgical hyperalgesia is a key driver of chronic pain transition [2]. Dex was administered twice daily on postoperative Days 1–2 (Figure 1G–J). Baseline PWMT measured prior to the first dose did not differ between SI + saline and SI + Dex groups (Figure 1H,I). To evaluate sustained effects independent of acute drug action, behavioral testing was performed 12 h after the final dose each day (i.e., immediately before the next scheduled injection) (Figure 1G). During the early postoperative phase (Days 2–3), Dex treatment significantly increased PWMT in SI mice, whereas no comparable effect was observed in incision‐only animals (Figure 1H). Given this short‐term benefit, we further assessed longer‐term outcomes. Longitudinal measurements on postoperative Days 7, 14, 21, and 28 showed that early Dex administration accelerated recovery of both mechanical thresholds and thermal latency in SI mice (Figure 1I,J). Together, these findings indicate that Dex exerts a sustained modulatory effect on stress‐potentiated pain, and that early postoperative administration can attenuate the progression toward prolonged SIH.

### 2.2 Local Administration of Dex Suppresses AIC Hyperactivity Under SIH Conditions

**FIGURE 2:**
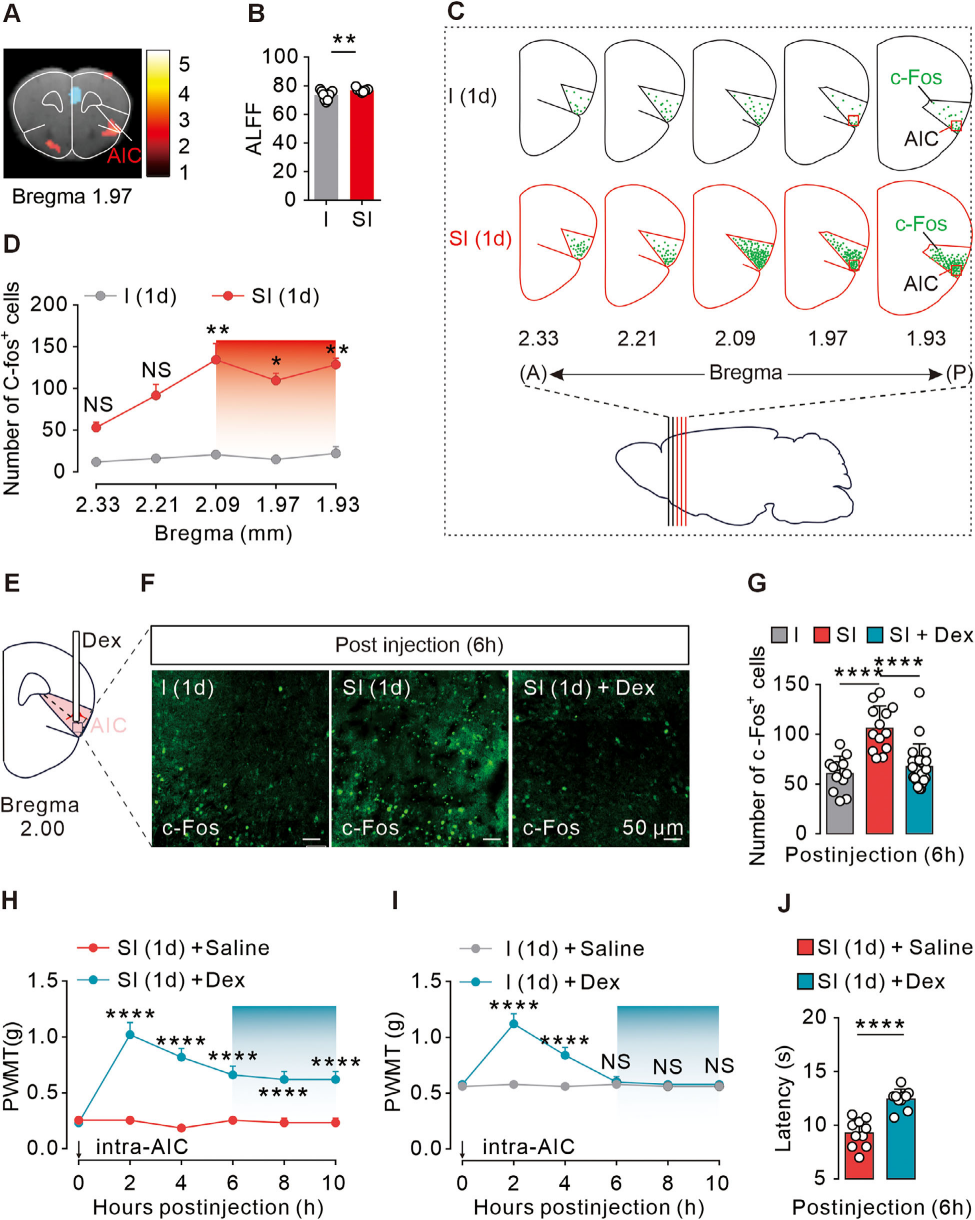
Local administration of Dex suppresses AIC activity and rapidly reverses hyperalgesia in SIH mice. (A) Representative fMRI image in the anterior insular cortex (AIC) in mice subjected to incision only (I) or stress + incision (SI). See also Figure S2. (B) Average amplitude of low‐frequency fluctuations (ALFF) changes in the anterior insular cortex (AIC) in mice subjected to incision only (I) or stress + incision (SI). *n* = 9 mice per group. (C) Reconstructed c‐Fos immunostaining from representative coronal brain slices containing the AIC. Green dots indicate c‐Fos^+^ cells (*n* = 6 slices from 6 mice). (D) Rostro‐caudal quantification of c‐Fos expression (*n* = 6 slices from 6 mice). (E) Schematic representation of right‐lateral cannula implantation targeting the AIC in SIH mice. (F) Representative images of c‐Fos expression in the AIC (bregma: +2.00 mm) following local infusion of Dex (12 µM, 500 nL) or saline (500 nL) into the right AIC in I or SI mice (*n* = 12 or 13 samples from 6 mice per group). (G) Quantification of c‐Fos expression in the AIC (bregma: +2.00 mm) following local infusion of Dex (12 µM, 500 nL) or saline (500 nL) into the right AIC in I or SI mice (*n* = 12 or 13 samples from 6 mice per group). (H) Contralateral PWMT was measured within 10 h after intra‐AIC saline or Dex administration to evaluate rapid antihyperalgesic effects in SI mice. *n* = 10 mice per group. (I) Contralateral PWMT after intra‐AIC saline or Dex administration in I mice. *n* = 10 mice per group. (J) Thermal withdrawal latency was measured at 6 h after intra‐AIC saline or Dex administration in SI mice. *n* = 10 mice per group. Data are presented as mean ± SEM. NS, no significant. *p < 0.05, **p < 0.01, ****p < 0.0001. Statistical analysis: two‐tailed unpaired *t*‐tests (B and J), two‐way repeated‐measures ANOVA with Bonferroni's posthoc test (D, H, and I), and one‐way ANOVA with Tukey's posthoc test (G). Source data are provided in the Supporting Information.

To identify the key brain regions involved in SIH, we first conducted resting‐state functional MRI (fMRI) to analyze the amplitude of low‐frequency fluctuations (ALFF), a widely used metric reflecting spontaneous neuronal activity at rest [22]. Scans were performed on group SI and group I mice on postoperative Day 1. Among 16 representative emotion and pain‐associated brain regions, including the AIC, prefrontal cortex (PFC), hippocampus, bed nucleus of the stria terminalis, orbitofrontal cortex (OFC), periaqueductal gray (PAG), dorsal raphe nucleus, and others [9, 23, 24, 25, 26], the most pronounced increase in ALFF was observed in the AIC of group SI mice, specifically in the 0.01‐Hz frequency band (Figures 2A,B and S2). This suggests that stress induces upregulation of spontaneous neuronal activity in the AIC. To validate this fMRI‐based observation, we performed immunofluorescence staining for c‐Fos, a marker of neuronal activation, in consecutive coronal sections of the AIC. Sections were anatomically aligned using the Allen Mouse Brain Atlas. Compared with group I mice, group SI mice exhibited significantly elevated c‐Fos expression in the AIC (Figure 2C,D), confirming that AIC neurons are activated by preoperative stress. These data implicate the AIC as a key brain region recruited during SIH and suggest that increased AIC activity contributes to pain hypersensitivity under stress conditions.

Next, we examined whether intra‐AIC microinjection of Dex could reduce this hyperactivity. A single unilateral infusion of Dex (12 µM, 500 nL) was delivered into the right AIC via guide cannula in group SI mice. Six hours postinfusion, c‐Fos staining revealed that local Dex administration significantly suppressed stress‐induced neuronal activation in the AIC (Figure 2E–G). To evaluate the functional relevance of this suppression, we performed behavioral pain assays following intra‐AIC Dex injection. At 6 h postinjection, mechanical pain threshold testing demonstrated that local infusion of Dex robustly reversed tactile hypersensitivity in group SI mice, with no notable effect in group I mice (Figure 2H,I). Similarly, hot plate testing showed that Dex significantly prolonged thermal withdrawal latencies in group SI mice (Figure 2J), indicating reduced thermal hypersensitivity. Taken together, these results demonstrate that the AIC is a critical node for SIH‐related pain amplification, and that intra‐AIC Dex administration effectively alleviates hyperalgesia by dampening AIC neuronal hyperactivity. These findings highlight the AIC as a key target for Dex‐mediated modulation of pain circuits. However, the specific neuronal subtypes mediating this effect remain to be identified.

### 2.3 Chemogenetic Inhibition of AIC^Glu^ Neurons Reproduces the Antihyperalgesic Effects Observed Following Dex Treatment

**FIGURE 3:**
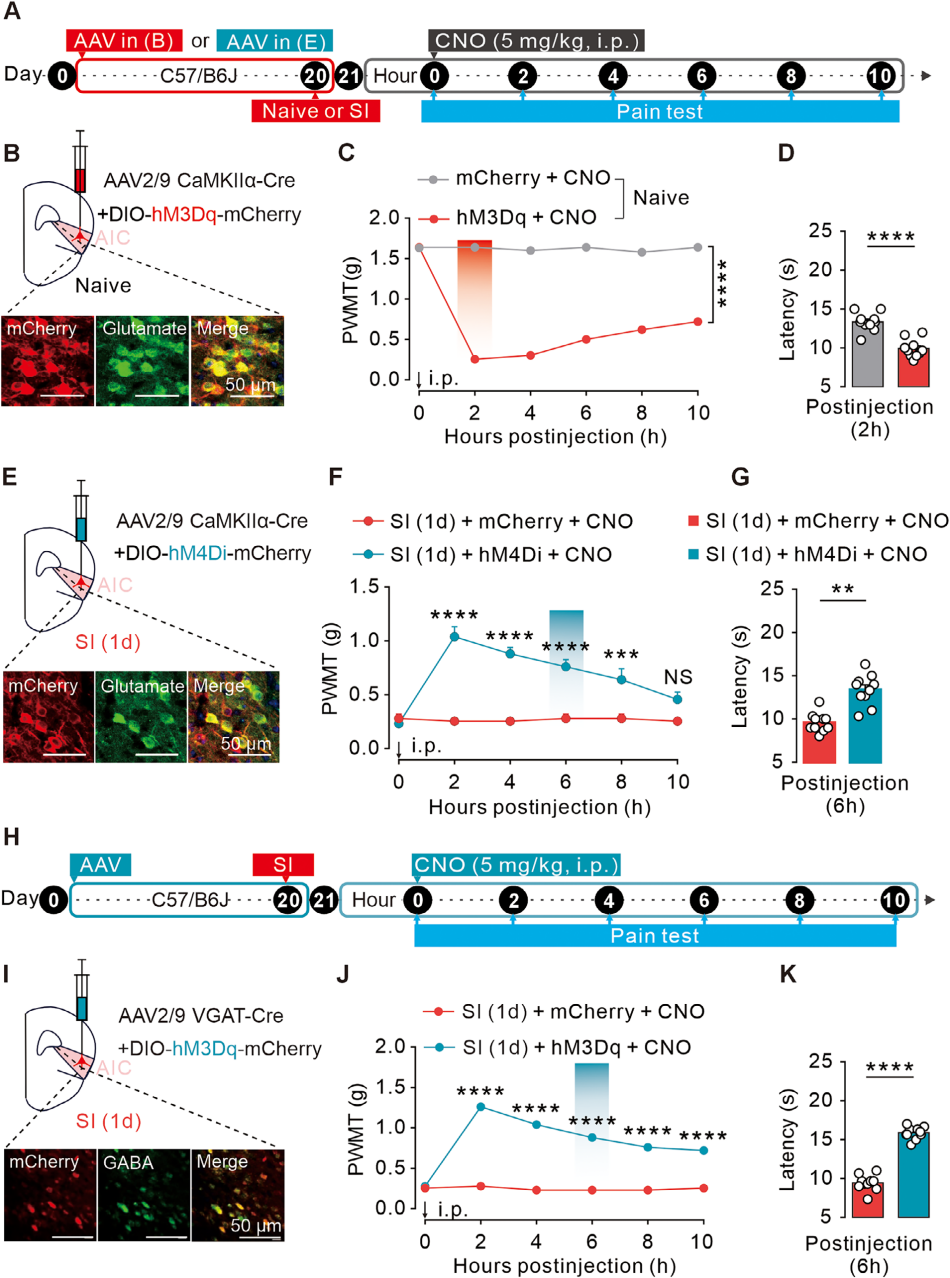
Chemogenetic inhibition of AIC^Glu^ or activation of AIC^GABA^ neurons mimics the antihyperalgesic effects of Dex. (A) Schematic representation of the experimental design for AAV infusion, clozapine‐N‐oxide (CNO) administration, and behavioral tests. SI, stress + incision. (B) Illustration of right‐lateral virus infusion targeting the anterior insula cortex (AIC) in naive mice. Pink shading indicates the anatomical location of the AIC. Representative images show mCherry^+^ neurons colabeled with glutamate in the right‐lateral AIC. (C) Contralateral paw withdrawal mechanical threshold (PWMT) was measured following chemogenetic activation of hM3Dq‐expressing AIC^Glu^ neurons via intraperitoneal CNO in naïve mice (*n* = 10). (D) Thermal withdrawal latency measured following chemogenetic activation of hM3Dq‐expressing AIC^Glu^ neurons via intraperitoneal CNO in naïve mice (*n* = 10). (E) Experimental schematic representation as in (B), but for chemogenetic inhibition in AIC^Glu^ neurons in SI mice (1 day postinjury). (F) Contralateral PWMT following chemogenetic inhibition of hM4Di‐expressing AIC^Glu^ neurons with intraperitoneal CNO in SIH mice (*n* = 10). (G) Thermal withdrawal latency following chemogenetic inhibition of hM4Di‐expressing AIC^Glu^ neurons with intraperitoneal CNO in SIH mice (*n* = 10). (H) Experimental schematic representation for chemogenetic activation of AIC^GABA^ neurons. (I) As presented in (B), but showing mCherry^+^ neurons colabeled with GABA in the right‐lateral AIC from SI mice (1 day postinjury). (J) Contralateral PWMT over 10 h following chemogenetic activation of hM3Dq‐expressing AIC^GABA^ neurons with intraperitoneal CNO in SIH mice (*n* = 10). Scale bar: 50 µm. (K) Thermal withdrawal latency at 6 h following chemogenetic activation of hM3Dq‐expressing AIC^GABA^ neurons with intraperitoneal CNO in SIH mice (*n* = 10). Scale bar: 50 µm. Data are presented as mean ± SEM. NS, no significant. **p < 0.01, ***p < 0.001, ****p < 0.0001. Statistical tests: two‐way repeated‐measures ANOVA with Bonferroni's posthoc test (C, F, and J) and two‐tailed unpaired *t*‐tests (D, G, and K). See also Supporting Information.

Glutamatergic and GABAergic neurons comprise the principal neuronal subtypes in the AIC. To explore their specific roles in pain modulation, we employed chemogenetic approaches to manipulate these populations in both naive and SI mice. We microinjected AAV2/9–EF1*α*–DIO–hM3Dq–mCherry or AAV2/9–EF1*α*–DIO–mCherry into the right AIC of naive mice, along with AAV2/9–CaMKII*α*–Cre to selectively drive Cre‐dependent expression in glutamatergic neurons, 21 days prior to pain tests (Figure 3A,B). Immunofluorescence analysis confirmed that 88.9% of mCherry^+^ cells coexpressed glutamate, validating specific targeting of excitatory neurons (Figure 3B). Following systemic administration of clozapine‐N‐oxide (CNO; 5 mg/kg, i.p.), mice exhibited a significant reduction in mechanical withdrawal threshold and thermal latency (Figure 3C,D), indicating that activation of AIC glutamatergic neurons enhances pain‐like behaviors.

To establish a causal link between AIC^Glu^ neuronal activity and SIH, and to determine whether their inhibition could replicate the analgesic effects of Dex, we employed a chemogenetic approach to selectively suppress AIC glutamatergic activity in SI mice. AAV2/9–CaMKII*α*–Cre was coinfused with either AAV2/9–EF1*α*–DIO–hM4Di–mCherry or control AAV2/9–EF1*α*–DIO–mCherry into the right AIC 21 days prior to intra‐AIC CNO administration and behavioral tests (Figure 3A,E). Immunofluorescence analysis confirmed that 94.0% of mCherry^+^ cells colocalized with glutamate staining (Figure 3E). Consistent with the effects observed following local Dex administration, chemogenetic inhibition of AIC^Glu^ neurons significantly reversed the reduced mechanical pain threshold and shortened thermal latency observed in SI mice (Figure 3F,G), indicating that directly suppressing AIC^Glu^ neuronal activity is sufficient to mimic the antihyperalgesic effects of Dex in stress‐induced postoperative pain.

To assess the role of inhibitory neurons, we injected AAV2/9–EF1*α*–DIO–hM3Dq–mCherry or AAV2/9–EF1*α*–DIO–mCherry into the right AIC of SI mice, along with AAV2/9–VGAT–Cre to target GABAergic neurons (Figure 3H,I). Immunofluorescence staining confirmed that 90.8% of mCherry^+^ cells colocalized with GABA (Figure 3I). Chemogenetic activating AIC GABAergic neurons caused a significant increase in mechanical threshold and thermal latency in SI mice (Figure 3J,K), indicating that GABAergic activity exerts antinociceptive effects under SIH conditions. Together, these results demonstrate that excitatory glutamatergic neurons in the AIC promote hyperalgesia‐like behaviors, whereas suppressing AIC^Glu^ neurons can mimic the antihyperalgesic effects observed with Dex treatment in SIH conditions.

### 2.4 Dex Suppresses the Hyperexcitability and Hypersensitivity of AIC^Glu^ Neurons in SI Mice

**FIGURE 4:**
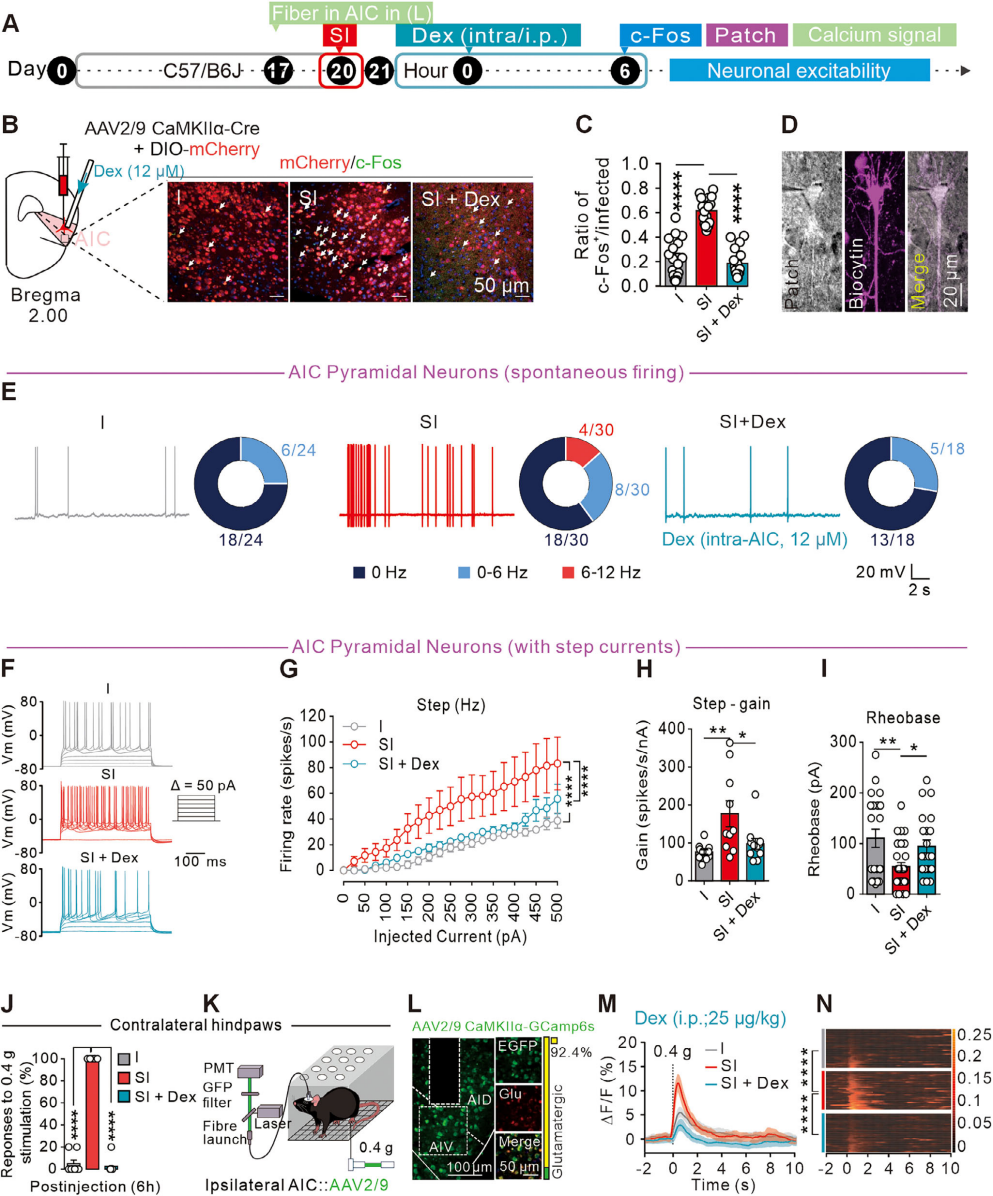
Dex sustainedly suppresses stress‐induced increases in spontaneous activity of AIC^Glu^ neurons and sensitivity to exogenous stimuli. (A) Experimental schematic representation for (B–H). SI, stress + incision. I, incision. Dex, dexmedetomidine. To investigate Dex's plastic effects on AIC neurons, patch clamp was performed at 6 h, if any, after Dex dosing. (B) Illustration of right‐lateral virus infusion and local Dex (12 µM, 500 nL) administration in the anterior insula cortex (AIC) for c‐Fos expression analysis in AIC^Glu^ neurons. White arrowheads indicate the mCherry^+^ glutamatergic neurons coexpressing c‐Fos. (C) Quantification of mCherry^+^ glutamatergic neurons coexpressing c‐Fos (*n* = 17, 18, and 18 samples for groups I, SI, or SI + Dex, respectively). (D) Morphology of biocytin‐labeled patched pyramidal neurons. Scale bar: 20 µm. (E) Representative current‐clamp recordings (left) and neuronal classification (right) based on spontaneous action potential (AP) frequency. (F) Example current‐clamp traces of APs evoked by step current injections (0 to +500 pA). (G) Quantification of AP firing rate (*n* = 9–11 cells from 3 to 6 mice per group). (H) Quantification of AP step gain (*n* = 10–11 cells from 3 mice per group). (I) Quantification of AP rheobase (*n* = 21–29 cells from 3 to 4 mice per group). (J) Mechanical withdrawal response to a 0.4‐g von Frey filament (*n* = 8 mice). (K) Schematic representation of fiber photometry recording in mice (*n* = 8 mice). (L) Representative images of fiber photometry recording in mice. Scale bars: 100 µm (left) and 50 µm (right) (*n* = 8 mice). (M) Mean Δ*F*/*F* (%) of AIC^Glu^ neuronal activity in response to 0.4‐g mechanical stimulation (subthreshold in group I). (N) Heatmaps showing 30 trials of Ca^2+^ responses per group in (M) (*n* = 30 trials from 8 mice). Data are shown as mean ± SEM. *p < 0.05, **p < 0.01, ***p < 0.001, ****p < 0.0001. Statistical tests: one‐way ANOVA with Tukey's posthoc test (C, H, J, and N), or with Holm–Sidak's posthoc test for (I), and two‐way ANOVA with Bonferroni's posthoc test (G). See also Supporting Information.

To determine whether Dex attenuates the spontaneous activity of AIC^Glu^, we evaluated c‐Fos expression in AIC glutamatergic neurons of mice from group I, SI, and SI + Dex. AIC^Glu^ neurons were selectively labeled through local injection of AAV2/9–EF1*α*–DIO–mCherry combined with AAV2/9–CaMKII*α*–Cre 21 days prior to c‐Fos staining. Local Dex was administered into the AIC via a preimplanted catheter on Day 1 postsurgery in SI mice (Figure 4A). Immunofluorescence staining revealed that Dex significantly reduced the proportion of c‐Fos^+^ cells among mCherry^+^ AIC neurons (Figure 4B,C). Furthermore, whole‐cell patch‐clamp recordings of AIC^Glu^ neurons, identified as pyramidal cells morphologically (Figure 4D), in acute brain slices showed that the proportion of neurons not firing spontaneously was significantly reduced in SI mice (18 of 30 cells, 60.0%), compared with group I (18 of 24 cells, 75.0%) (Figure 4E). Notably, Dex treatment enhanced this proportion in SI + Dex mice (13 of 18 cells, 72.2%) (Figure 4E). In addition, the percentages of neurons with a higher discharge frequency (6–12 Hz) in group SI, I, and SI + Dex were 13.3% (four of 30 cells in group SI), 0% (0 of 24 cells in group I), and 0% (0 of 18 cells in group SI + Dex), respectively (Figure 4E), indicating that Dex suppresses stress‐induced AIC neuronal hyperactivity.

We then compared the characteristics of typical spontaneous action potentials (APs) and found that the AP firing trajectories of the SI group were significantly different from those of the I or Dex + SI groups (Figure S3A–D). Although basic membrane properties showed no significant differences in membrane capacitance or resistance among groups (Figure S3E,F), SI mice exhibited a markedly depolarized resting membrane potential (RMP) compared with controls (−52.75 ± 2.18 mV vs. −62.98 ± 3.15 mV) (Figure S3G), along with a depolarized firing threshold (Figure S3H). The reduced difference between RMP and firing threshold in SI mice (−6.04 ± 0.85 mV vs. −10.90 ± 1.49 mV) (Figure S3I) indicated an increased neuronal excitability. These alterations were significantly normalized by Dex treatment, with both RMP and the difference between RMP and firing threshold returning to the levels observed in group I (Figure S3G,I).

Further analysis of AP properties revealed no significant differences in amplitude across groups (Figure S3J). However, SI mice displayed a shorter half‐duration (Figure S3K), reduced rise time (Figure S3L), and steeper maximum rise and decay slopes of APs (Figure S3M,N), indicating an increased firing frequency after preoperative stress. Dex treatment reversed the shortened half‐duration and the heightened rise slope of APs in SI mice, though other AP parameters remained unchanged (Figure S3K–N). These results suggest that Dex effectively suppressed stress‐induced hyperexcitability of AIC^Glu^ neurons, supporting its role in reversing central sensitization mechanisms underlying SIH.

To assess whether Dex modulates pain hypersensitivity specifically in AIC^Glu^ neurons of SI mice, we examined both their intrinsic excitability and calcium responses to a subthreshold mechanical stimulus (Figure 4A). Whole‐cell patch‐clamp recordings using a stepwise current injection protocol revealed that AIC^Glu^ neurons in SI mice exhibited significantly increased firing rates (Figure 4F,G), enhanced step‐gain (slope of firing rate vs. injected current, reflecting neuronal sensitivity; Figure 4H), and reduced rheobase (minimal current to evoke an AP; Figure 4I) relative to incision‐only (I) mice. Notably, these hyperexcitability features were effectively normalized following Dex administration (Figure 4F–I). Complementary recordings using ramped current injections (Figure S4A,B) further showed that SI mice displayed elevated ramp gain and shorter ramp latency (Figure S4C,D), reflecting heightened responsiveness to gradually increasing inputs. AIC^Glu^ neurons in SI mice also exhibited trends toward impaired adaptation, as indicated by modestly reduced ramp‐adaptation and increased adaptation ratio (Figure S4E,F), suggesting compromised sensory filtering. All of these alterations were significantly restored by Dex treatment (Figure S4C–F).

To probe functional relevance in vivo, we applied a 0.4‐g von Frey filament, which elicited a 100% paw‑withdrawal in SI mice but minimal responses in I or SI + Dex mice (Figure 4J), indicating SIH to an otherwise innocuous stimulus. Calcium imaging using fiber photometry in freely behaving mice, after targeted AAV2/9–CaMKII*α*–GCaMP6s expression in the right AIC (Figure 4K), confirmed this effect. Immunofluorescence analysis showed that 92.4% of EGFP‐labeled neurons coexpressed glutamate (Figure 4L), validating specific targeting of AIC^Glu^ neurons. In response to the 0.4‐g stimulus, SI mice exhibited significantly elevated calcium transients, which were effectively reversed by systemic Dex treatment (Figure 4M,N).

To determine whether Dex also affects inhibitory AIC circuits, we examined AIC^GABA^ neurons using the same approaches (Figure S5A). Stepwise current injection revealed comparable firing rates (Figure S5B–D), step‐gain (Figure S5E), and rheobase (Figure S5F) across I, SI, and SI + Dex groups. Consistent results were observed in vivo: calcium transients in AIC^GABA^ neurons, monitored via AAV2/9–VGAT–GCaMP6s expression, were similar across groups in response to the 0.4‐g filament (Figure S5G–J). These findings indicate that Dex selectively modulates hyperexcitable AIC^Glu^ neurons in SIH without directly altering AIC^GABA^ neuronal activity.

Collectively, these results provide strong in vitro and in vivo evidence that Dex treatment reverses the increased sensitivity of AIC^Glu^, but not AIC^GABA^, neurons induced by preoperative stress, supporting its therapeutic potential in mitigating stress‐enhanced postoperative hyperalgesia.

### 2.5 Dex Restores the Excitatory–Inhibitory Balance of AIC^Glu^ Neurons in SI Mice

Like most cortical projection neurons [27], AIC^Glu^ neurons receive both excitatory and inhibitory synaptic inputs. The balance between these inputs plays a critical role in regulating neuronal excitability and sensitivity [28]. Therefore, we aimed to determine: (1) whether stress‐induced hyperactivity in AIC^Glu^ neurons was associated with an excitatory–inhibitory imbalance and (2) whether Dex treatment could restore this synaptic balance under SIH conditions.

**FIGURE 5:**
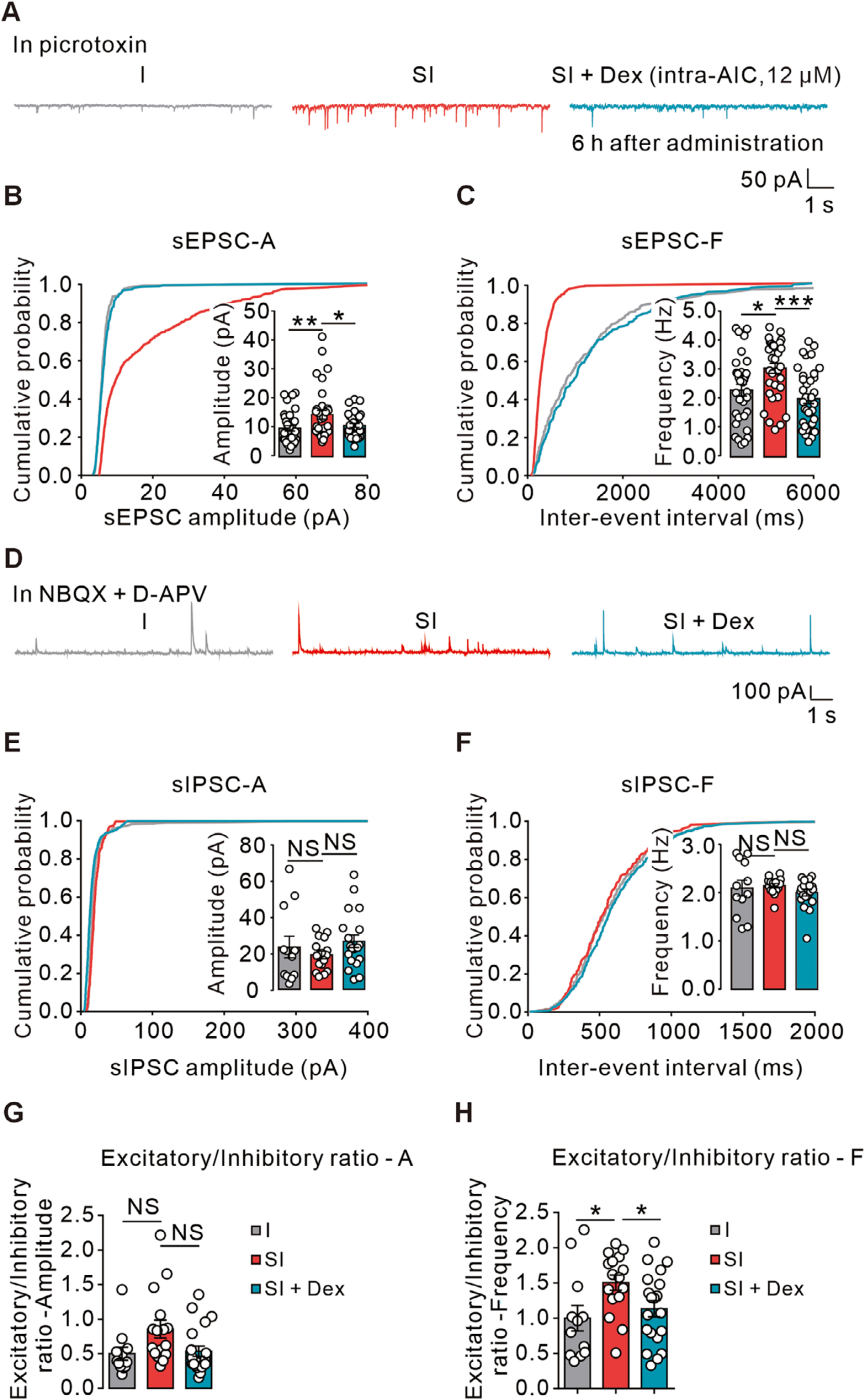
Local Dex treatment sustainably restores stress‐induced alterations in the excitatory/inhibitory ratio in the AIC. (A) Representative traces of spontaneous excitatory postsynaptic currents (sEPSCs) recorded, at 6 h after single‐dose administration if the drug was given, from anterior insula cortex (AIC) pyramidal neurons in stress + incision (SI, Day 1) mice with or without local dexmedetomidine (Dex; 12 µM) infusion. Incision (I, Day 1) mice served as controls for the SI (Day 1) group. (B) Cumulative probability plots of sEPSC amplitude (*n* = 33–36 cells from 4 to 6 mice per group) for neurons shown in (A). (C) Cumulative probability plots of sEPSC interevent interval (*n* = 34–36 cells from 4 to 6 mice per group) for neurons shown in (A). (D) Representative traces of sIPSCs recorded from AIC pyramidal neurons. (E) Cumulative probability plots of sIPSC amplitude (*n* = 12–20 cells from 3 to 5 mice per group) for neurons shown in (D). (F) Cumulative probability plots of sIPSC interevent interval (*n* = 12–20 cells from 3 to 5 mice per group) for neurons shown in (D). (G) Ratios of sEPSC to sIPSC frequency analyzed from (B and E) (*n* = 12–20 cells from 3 to 5 mice per group). (H) Ratios of sEPSC to sIPSC amplitude analyzed from (C and F) (*n* = 12–20 cells from 3 to 5 mice per group). Data are shown as mean ± SEM. NS, no significant. *p < 0.05, **p < 0.01, ***p < 0.001. Statistical tests: one‐way ANOVA with Tukey's posthoc test (B, C, and E–G) and one‐way ANOVA with Holm–Sidak's posthoc test (H). See also Supporting Information.

To evaluate the plasticity‑modulating, rather than acute, effects of Dex on synaptic transmission in AIC glutamatergic neurons, electrophysiological recordings were conducted 6 h after systemic (i.p.) Dex injection, at a time point when its acute analgesic effect had dissipated. We performed whole‐cell patch‐clamp recordings of spontaneous excitatory postsynaptic currents (sEPSCs) and inhibitory postsynaptic currents (sIPSCs) in the same AIC^Glu^ neurons. Recordings were performed at holding potentials of −70 mV for sEPSCs (Figure 5A–C) or 0 mV for sIPSCs (Figure 5D–F), allowing calculation of the excitatory/inhibitory ratio based on both amplitude and frequency (Figure 5G,H). Compared with mice in group I, preoperative stress significantly increased both the amplitude and frequency of sEPSCs in AIC^Glu^ neurons from SI mice (Figure 5B,C). These increases were completely reversed by local Dex infusion (Figure 5B,C), indicating that Dex effectively reduced the heightened excitatory drive under SI conditions. As for inhibitory inputs, the amplitude or frequency of sIPSCs remained unchanged either between SI and I groups, or between SI + Dex and SI groups (Figure 5E,F), underlining again the critical contribution of excitatory drive in the development of SIH, and the selective suppression of excitatory drive to AIC^Glu^ by Dex treatment.

Importantly, further analysis of excitatory/inhibitory ratio revealed that stress selectively elevated the sEPSC/sIPSC frequency ratio, without affecting the amplitude ratio (Figure 5G). This shift highlights the crucial role of enhanced presynaptic excitatory inputs in driving hyperexcitability in AIC^Glu^ neurons after stressor exposure. Notably, Dex infusion restored the excitatory/inhibitory frequency ratio to baseline levels (Figure 5H), suggesting that its analgesic effect depends on rebalancing presynaptic excitatory and inhibitory inputs onto AIC^Glu^ neurons.

### 2.6 OFC–AIC Glutamatergic Circuit Activation Contributes to SIH

**FIGURE 6:**
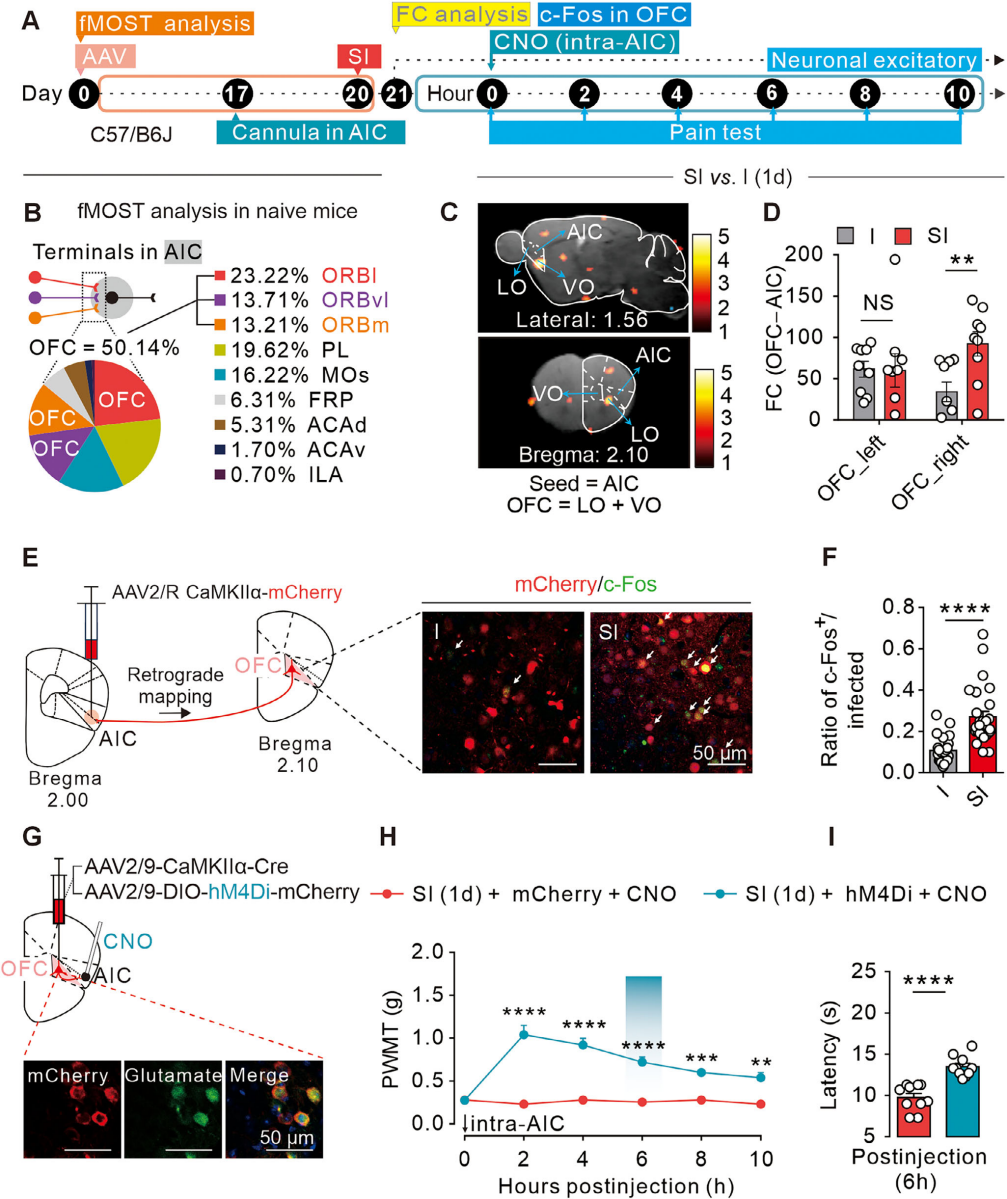
Chemogenetic inhibition of the OFC^Glu^–AIC circuit alleviates hyperalgesia‐like behaviors in SI mice. (A) Schematic representation of the experimental design for (B–I). AIC, anterior insula cortex. SI, stress + incision. FC, functional connection. OFC, orbitofrontal cortex. CNO, clozapine‐N‐oxide. (B) fMOST analysis showing major AIC inputs from indicated anterior brain regions in naïve C57/B6J mice, based on an open‐source projectome atlas from WT C57/BL6 mice (available at https://mouse.digital‐brain.cn/projectome). ORBI (OFC), orbital area. PL, prelimbic area. MOs, secondary motor area. ORBvl, ventrolateral part of the orbital area. ORBm, medial part of the orbital area. FRP, frontal pole. ACAd, dorsal part of the anterior cingulate area. ACAv, ventral part of the anterior cingulate area. ILA, infralimbic area. (C) Representative sagittal and coronal images from FC fMRI analysis. I, incision. See also Figure S6. (D) Quantification of data in (C) (D, *n* = 10 or 9 mice for group I or SI, respectively). (E) Illustration of right‐lateral retrograde labelling virus infusion in the anterior insula cortex (AIC) for c‐Fos expression analysis in OFC^Glu^ neurons. White arrowheads indicate the mCherry^+^ glutamatergic neurons coexpressing c‐Fos. (F) Quantification of mCherry^+^ glutamatergic neurons coexpressing c‐Fos (*n* = 29 areas from 6 mice per group). (G) Right‐lateral viral infusion into the OFC and cannula implantation in the AIC for chemogenetic inhibition of the OFC^Glu^–AIC circuit in SI (Day 1) mice. Representative images show mCherry^+^ glutamatergic neurons in the right‐lateral OFC. Scale bars: 50 µm. (H) Contralateral PWMT following chemogenetic inhibition of the OFC^Glu^–AIC circuit by intra‐AIC injection of CNO (5 µM) in SI mice. *n* = 10 mice per group. (I) Thermal withdrawal latency following chemogenetic inhibition of the OFC^Glu^–AIC circuit by intra‐AIC injection of CNO (5 µM) in SI mice. *n* = 10 mice per group. Data are shown as mean ± SEM. NS, no significant. **p < 0.01, ***p < 0.001, ****p < 0.0001. Statistical tests: two‐tailed unpaired *t*‐tests (D, F, and I) and two‐way ANOVA with Bonferroni's posthoc test (H). See also Supporting Information.

The results above revealed that Dex treatment attenuates excessive glutamatergic inputs onto AIC^Glu^ neurons via presynaptic inhibition. However, the precise upstream source of these excitatory inputs and the underlying mechanisms remain unclear. Previous studies have identified several brain regions projecting glutamatergic afferents to the AIC, including the PFC, OFC, anterior cingulate cortex (ACC), ventral tegmental area (VTA), basolateral amygdala (BLA), parabrachial nucleus, and LC [8, 29]. To anatomically assess their relative contributions to SIH, we analyzed axon input from these regions into the AIC using an online fluorescence micro‐optical sectioning tomography (fMOST) database (https://mouse.digital‐brain.cn Figure 6A). Quantitative analysis revealed that OFC‐originating axonal projections accounted for the majority of input to the AIC (orbital area [ORBI]: 23.22%, ventrolateral part of orbital area [ORBvl]: 13.71%, medial part of orbital area [ORBm]: 13.21%; 50.14% in total) (Figure 6B). To explore functional connectivity (FC), resting‐state fMRI was performed in SI and I mice using the right AIC as the seed region. The analysis revealed significantly increased FC between the OFC and AIC in SI mice (Figures 6A,C,D and S6). To interrogate circuit‑specific activity, we employed a retrograde viral strategy to label CaMKIIα‑expressing OFC neurons projecting to the AIC, combined with c‑Fos immunostaining. Compared with group I, SI mice exhibited a markedly higher proportion of c‑Fos‑positive neurons within this projection‑defined OFC–AIC population (Figure 6A,E,F), indicating that enhanced activity of the OFC–AIC glutamatergic circuit contributes to the development of SIH.

To confirm the anatomical existence of a direct OFC–AIC glutamatergic pathway, we performed anterograde transsynaptic tracing via injection of AAV2/1–CaMKII*α*–Cre into the OFC of Cre‐dependent *Td‐tomato reporter mice* (Figure S7A). Labeled AIC neurons exhibited strong colocalization with glutamate staining, confirming a direct OFC–AIC glutamatergic circuit. Complementary retrograde tracing with AAV2/R–CaMKII*α*–Cre injected into the AIC of *wild‐type mice* further revealed mCherry‐labeled, glutamate‐positive neurons in the OFC, which were also well colabeled with glutamate staining (Figure S7B,C), supporting a direct OFC^Glu^–AIC^Glu^ circuit.

To determine the electrophysiological properties of this OFC^Glu^–AIC^Glu^ circuit, we conducted optogenetically assisted patch‐clamp recordings. ChR2 was selectively expressed in OFC^Glu^ neurons by coinjecting AAV2/9–CaMKII*α*–Cre and AAV2/9–DIO–ChR2–mCherry viruses (Figure S8A,B). Upon blue light stimulation of OFC axonal terminals in the AIC, inward EPSCs were observed in AIC pyramidal neurons and were completely abolished by NBQX (20 µM) (Figure S8C1,C2), confirming *α*‐amino‐3‐hydroxy‐5‐methyl‐4‐isoxazole propionic acid (AMPA)‐mediated excitatory transmission. The average latency of light‐evoked EPSCs was 5.19 ± 0.24 ms (Figure S8C3), consistent with previously reported monosynaptic delays [30]. Tetrodotoxin (TTX; 0.5 µM) completely blocked photostimulation‐evoked inward currents, which were subsequently rescued by 4‐aminopyridine (4‐AP; 300 µM) (Figure S8D), further supporting monosynaptic glutamatergic transmission.

To establish a causal relationship between the activation of OFC^Glu^–AIC^Glu^ circuit and SIH, we performed chemogenetic inhibition of OFC^Glu^ terminals in the AIC, AAV2/9–CaMKII*α*–Cre was coinfused with either AAV2/9–EF1*α*–DIO–hM4Di–mCherry or AAV2/9–EF1*α*–DIO–mCherry into the OFC 21 days prior to local injection of CNO through a guided cannula in AIC (Figure 6A,G). SIH was induced 1 day before CNO administration. Immunofluorescence analysis confirmed strong colocalization of mCherry and glutamate staining in the OFC (Figure 6G). Behavioral testing revealed that chemogenetic inhibition significantly alleviated mechanical and thermal hypersensitivity under SIH conditions, as shown by improved PWMT and increased latency in the hot plate test, compared with SI + mCherry controls (Figure 6H,I). These findings collectively indicate that the OFC–AIC glutamatergic circuit is a critical contributor to SIH and that its hyperactivation plays a causal role in the expression of pain hypersensitivity following stress exposure.

### 2.7 Dex Alleviates Hyperalgesia‐Like Behaviors by Suppressing Excessive Excitatory Transmission at OFC–AIC Synapses in an α2‐AR‐Dependent Presynaptic Manner

**FIGURE 7:**
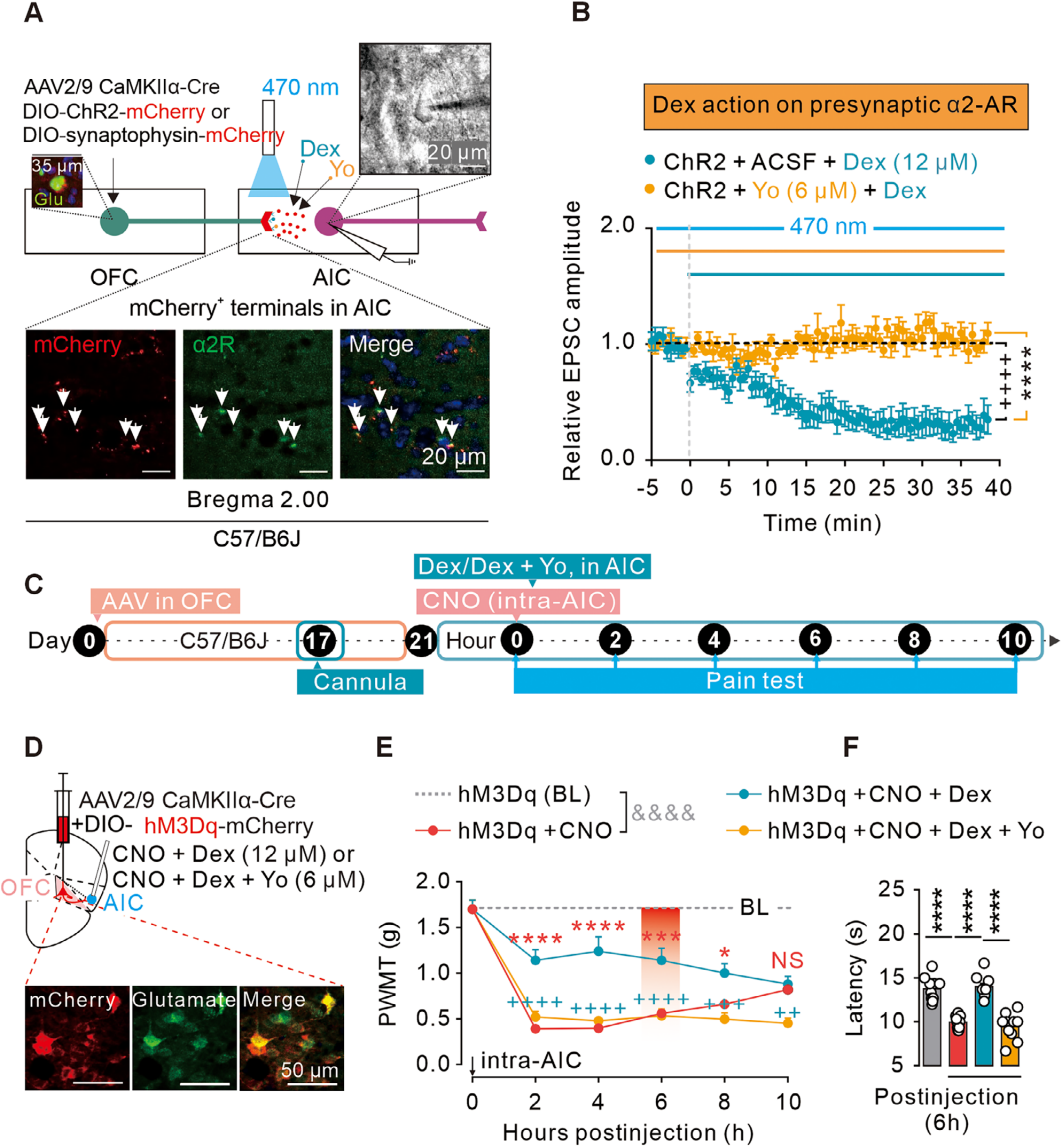
Local Dex infusion alleviates hyperalgesia‐like behaviors by suppressing excessive excitatory transmission at OFC–AIC synapses in an *α*2‐AR‐dependent manner. (A) Schematic representation of AAV infusion into the orbitofrontal cortex (OFC), drug delivery, and patch clamp recordings in ex vivo brain slices containing the anterior insula cortex (AIC). Dex, dexmedetomidine. Yo, yohibine. Channelrhodopsin 2 (ChR2)–mCherry (red) and *α*2‐adrenergic receptor (*α*2‐AR; green) expression in the AIC are shown. (B) Instant normalized light‐evoked EPSCs before and after an antihyperalgesic dose of Dex application (10 min, 12 µM) in the presence of Yo (10 min, 6 µM) or vehicle (*p* < 0.0001; *n* = 4–6 cells from 4 to 6 mice per group). Colored bars indicate the timing of light stimulation (blue), Dex application (green), and Yo application (yellow). Data are shown as mean ± SEM. ^++++^ *p* < 0.0001 vs. baseline; ****p < 0.0001 vs. Yo + Dex in group ACSF + Dex. Two‐tailed unpaired *t*‐tests was used. (C) Experimental design schematic. CNO, clozapine‐N‐oxide. (D) Right‐lateral chemogenetic activation of the OFC^Glu^–AIC circuit in the indicated groups. Representative images show mCherry^+^ glutamatergic neurons in the right‐lateral OFC. Scale bars: 50 µm. (E) Contralateral PWMT after intra‐AIC injection of Dex (12 µM) with Yo (6 µM) or Dex alone, during chemogenetic activation of the OFC^Glu^–AIC circuit by CNO (5 µM). *n* = 10 mice per group. (F) Thermal withdrawal latency after intra‐AIC injection of Dex (12 µM) with Yo (6 µM) or Dex alone, during chemogenetic activation of the OFC^Glu^–AIC circuit by CNO (5 µM). *n* = 10 mice per group. Data are shown as mean ± SEM. NS, no significant. ^&&&&^ *p* < 0.0001 vs. baseline (BL) in CNO‐injected mice; ****p < 0.0001 vs. CNO‐injected mice in (CNO + Dex)‐injected mice; ^++++^ *p* < 0.0001 vs. (CNO + Dex)‐injected mice in (CNO + Dex + Yo)‐injected mice. Statistical tests: Two‐way ANOVA with Bonferroni's posthoc test (E) and one‐way ANOVA with Tukey's posthoc test (F). See also Supporting Information.

Dex, a selective *α*2‐AR agonist, is well established to act presynaptically to suppress transmitter release. To determine whether Dex exerts its analgesic effects by inhibiting excessive excitatory transmission at the OFC–AIC synapse, we first assessed *α*2‐AR expression at OFC axon terminals within the AIC. Immunofluorescence analysis revealed substantial colocalization of mCherry‐labeled OFC terminals with *α*2‐AR staining in the AIC (Figure 7A), indicating the presynaptic localization of *α*2‐ARs at these glutamatergic terminals. To test whether Dex‐mediated modulation of this circuit depends on *α*2‑AR activation, we optogenetically stimulated ChR2‐expressing OFC terminals in the AIC to mimic excessive excitatory input, while recording light‐evoked EPSCs in AIC pyramidal neurons (Figure 7B; viral strategy as in Figure S8B). Whole‐cell voltage‐clamp recordings demonstrated that Dex significantly suppressed light‐evoked EPSCs following 40 min of blue light stimulation. Importantly, this inhibitory effect was completely reversed by coapplication of yohimbine (6 µM) (Figure 7B), indicating that Dex reduces excitatory synaptic transmission at OFC–AIC synapses through an *α*2‐AR‐dependent presynaptic mechanism.

To further validate these findings at the behavioral level, we examined whether Dex can modulate hyperalgesia‐like behaviors induced by activation of the OFC–AIC circuit. We employed chemogenetic activation of OFC^Glu^ neurons via excitatory hM3Dq, with local CNO injection into the AIC (Figure 7C,D). Immunostaining confirmed a robust colocalization (90.2%) of the mCherry signal with glutamate in OFC neurons (Figure 7D). Behavioral assays revealed that CNO microinjection led to a significant reduction in both PWMT and hot‐plate latency compared with baseline (Figure 7E,F), indicating that activation of the OFC–AIC circuit was sufficient to induce hyperalgesia‐like behaviors reminiscent of those observed in SIH. Notably, local administration of Dex effectively reversed these hyperalgesia‐like behaviors. However, coadministration of yohimbine completely abolished the analgesic effects of Dex (Figure 7E,F). Consistent with our circuit‐mapping results (Figure 6C–F), we further examined the dependence of Dex's effect on *α*2‑ARs in SI and I mice. Local Dex administration in the AIC significantly elevated mechanical and thermal thresholds in SI mice, and these effects were blocked by yohimbine (Figure S9A–C), whereas no significant changes were observed in group I mice. Overall, these results demonstrate that Dex alleviates hyperalgesia‐like and SIH behaviors induced by the excessive glutamatergic transmission in the OFC–AIC circuit through a presynaptic, *α*2‐AR‐dependent mechanism.

## 3 Discussion

Perioperative hyperalgesia is frequently accompanied by affective disturbances that exacerbate pain perception [31, 32]. This study identifies the AIC as a stress‐sensitive hub that integrates OFC glutamatergic inputs and mediates SIH following surgery. We demonstrate that stress exposure leads to hyperactivity in AIC glutamatergic neurons, driven by both enhanced presynaptic excitatory transmission from OFC and elevated intrinsic excitability and sensitivity of AIC pyramidal neurons. Critically, Dex effectively restores both synaptic and cellular maladaptations in this circuit, alleviating SIH via presynaptic *α*2‐AR‐dependent inhibition. These findings improve our understanding of how emotional stress modulates pain sensitivity through defined cortico‐cortical circuits.

While various brain regions, such as the PFC [23], ACC [33, 34], amygdala [35], and PAG [36, 37], have been implicated in stress‐related pain modulation, the precise neural substrates for SPS‐induced postoperative hyperalgesia remain unclear. Using fMRI‐guided circuit mapping, combined with c‐Fos activity profiling, we highlight the AIC as a key integrative hub for processing convergent emotional and nociceptive signals. This aligns with recent anatomical evidence that the AIC receives multimodal afferents [38, 39], and is well positioned to modulate pain perception in emotionally salient contexts. Glutamatergic neurons in the AIC are known to be implicated in various physiological and pathological processes, including drug addiction [8], reward‐related contextual memory [40], pain perception [9], social affective behavior [41], and compulsive behavior [42]. Functionally, we illustrate that AIC glutamatergic, but not GABAergic, neurons are essential for the development of SIH. Stress enhanced spontaneous firing and disrupted the excitatory/inhibitory balance within AIC microcircuits. These changes were causally linked to hyperalgesia, as chemogenetic activation of AIC glutamatergic neurons, whereas inhibition of glutamatergic neurons reduced pain responses. This supports a model in which stress skews AIC glutamatergic neurons toward pathological hyperexcitability.

At the synaptic level, we demonstrate that stress selectively enhances presynaptic excitatory drive to AIC glutamatergic neurons. Previous studies have identified multiple brain regions providing glutamatergic input to the AIC, including the PFC, OFC, ACC, and BLA [29]. Consistent with this, our seed‐based fMRI analysis revealed significantly enhanced FC between the AIC and the OFC, ACC, and amygdala in the SIH model. Among these, the OFC–AIC connection exhibited the most pronounced increase, suggesting a particularly important role. Anatomical data from the fMOST database further support this notion, showing that OFC projections account for over 50% of excitatory input to the AIC. Although whole‐region fMRI did not detect significant changes in OFC activity, circuit‐specific retrograde tracing combined with c‐Fos staining confirmed that SIH selectively activates OFC neurons projecting to the AIC. Most importantly, chemogenetic inhibition of the OFC–AIC pathway robustly alleviated SIH, establishing its causal contribution. While these results highlight the OFC–AIC circuit as a key pathway, we acknowledge potential contributions from other stress‐ and pain‐related networks. The ACC and amygdala are well‐established hubs in affective and sensory pain processing [43]. Stress induces synaptic potentiation in the ACC, which contributes to the emotional‐affective dimension of hyperalgesia [44]. Similarly, the amygdala, particularly the BLA and CeA, integrates stress and nociceptive signals, often exhibiting hyperexcitability in stress models [45, 46]. The enhanced ACC–AIC and amygdala–AIC FC observed in our fMRI screen may thus represent complementary or modulatory components of the stress‐pain network, with their specific synaptic and cellular mechanisms in SIH to be elucidated in future studies.

Despite the availability of perioperative analgesics, conventional treatments remain inadequate for managing hyperalgesia [16, 17, 47]. In the present study, Dex produced rapid analgesia within 4 h, consistent with its pharmacokinetics. Notably, while its acute analgesic effect on incision pain subsided by 6 h, a sustained antihyperalgesic effect against SIH persisted, indicating that Dex modulates not only acute nociception but also the underlying plasticity of prolonged hyperalgesia. Supporting this, systemic or local Dex administration normalized the excitatory/inhibitory balance and intrinsic excitability of AIC glutamatergic neurons when assessed 6 h postinjection, well beyond the acute analgesic window. Early short‐term Dex treatment (twice daily for 2 days postincision) effectively prevented the transition to persistent SIH. Together, these findings suggest that Dex exerts prolonged antihyperalgesic effects via plasticity‐dependent mechanisms, with the OFC–AIC circuit serving as a critical substrate. Importantly, while SPS alone induces SIH lasting approximately 14 days, the combination of SPS with incision (SI) produces more severe and prolonged allodynia lasting beyond 28 days, underscoring the clinical relevance of our model. Dex's preventive effect likely involves interference with the unique plasticity triggered by stress–nociceptive interactions. Whether mechanisms underlying “pure stress‐induced SIH” and “stress‐potentiated chronic postsurgical pain” substantially overlap, and whether Dex efficacy is specific to the latter, remain important questions for future investigation aimed at developing more targeted therapies.

At the molecular level, the antihyperalgesic effect of Dex is primarily mediated by *α*2‐ARs. In the present study, we found that *α*2‐ARs are expressed on presynaptic terminals of the OFC–AIC glutamatergic pathway, and that Dex suppresses excitatory synaptic transmission in this circuit via these receptors. Local *α*2‐AR blockade in the AIC abolished Dex's antihyperalgesic effect under circuit‐activated or SIH conditions, underscoring the necessity of *α*2‐AR signaling in this region. Although Dex can also act via other mechanisms, such as activating potassium channels [48] or modulating dopamine D2‑like receptors [49] in different contexts, our findings highlight the critical role of presynaptic *α*2‑ARs within the OFC–AIC pathway in mediating antihyperalgesic effects in SIH. Whether *α*2‑ARs are similarly expressed or engaged in other stress‑ or pain‑related circuits converging on the AIC, or at postsynaptic sites within AIC neurons, remains to be explored. Future studies combining circuit‑specific receptor mapping, conditional genetics, and detailed synaptic physiology will clarify the molecular architecture through which Dex modulates the OFC–AIC circuit and potentially other nodes of the stress–pain network.

These mechanistic insights carry important translational implications. First, our results underscore the value of early intervention: short‐term postoperative Dex administration can prevent the transition from acute SIH to persistent pain, supporting its use as a disease‐modifying strategy for patients at risk of chronic postsurgical pain. Second, identification of the OFC–AIC circuit as a key substrate for Dex's action suggests avenues for precise, personalized interventions. While systemic Dex use may be limited by side effects such as bradycardia and hypotension, the circuit‐specific mechanism revealed here opens the possibility of noninvasive neuromodulation approaches, such as repetitive transcranial magnetic stimulation [50], transcranial direct current stimulation [51], or transcranial focused ultrasound [52], that induce lasting, plasticity‐like changes in defined circuits [53]. Such strategies could offer circuit‐targeted analgesia without systemic side effects, personalizing treatment and broadening therapeutic options.

In conclusion, this study identifies a previously unrecognized OFC–AIC glutamatergic circuit as a key pathway in SIH, through synergistic enhancement of presynaptic glutamate release and postsynaptic excitability in AIC glutamatergic neurons. We further establish that *α*2‐AR‐mediated modulation by Dex is a potent, circuit‐targeted intervention that restores synaptic and intrinsic homeostasis, reducing hyperalgesia. These findings not only provide mechanistic insight into the neural basis of SIH but also highlight actionable targets for perioperative pain management.

## 4 Methods

### 4.1 Animals

All the experiments conducted in this study were approved by the Care Committee of Affiliated Drum Tower Hospital, Medical School of Nanjing University (license No. 2023AE01062). All animal experiments were performed strictly according to local and national guidelines to minimize animal suffering. Male C57BL/6J mice aged 8–9 weeks were obtained from Weitong Lihua Co., Ltd. (Beijing, China) and were group‐housed with 4–6 mice per cage unless a cannula or single optical fiber was implanted. The *Ai14 Td‐tomato reporter mice* were obtained from Southern Mode Organisms Co., Ltd. (Shanghai, China) and employed for monosynaptic anterograde tracing (Figure S7A). All housing conditions were maintained under controlled settings, including a 22 ± 1.0°C ambient temperature, 50% humidity, and a 12‐h light‐dark cycle (7:30 AM to 7:30 PM). Water and chow food were available to the mice ad libitum. All animals (weighing 25–30 g) were given 2 weeks to acclimate to the new environment before undergoing stress or surgery.

### 4.2 Single Prolonged Stress

The SPS model was established by slightly modifying previous models [54, 55]. Briefly, animals were first immobilized in ventilated 50‐mL polypropylene centrifuge tubes for 4 h and then subjected to forced swimming for 15 min in water at 25°C. Afterward, the animals were given a 15‐min rest in their home cages before being exposed to isoflurane until they were unconscious. Finally, mice underwent plantar electrical stimulation in a dark chamber, involving unconditioned foot shocks (1 mA for 4 s). To confirm successful induction of SIH, we applied strict inclusion criteria: only SI mice whose mechanical withdrawal thresholds decreased to 50–60% or less of their individual baseline (measured prior to SIH induction) were included in subsequent experiments [56].

### 4.3 Plantar Incisional Pain Model

The right plantar incision was performed as described in previous reports [16, 57]. Mice were completely anesthetized with isoflurane 1–3%. A 5‐mm longitudinal incision was executed on the plantar aspect of the right hindpaw, through which the skin, fascia, and muscle were opened layer by layer. The wound was then gently pressed for 60 s to ensure bleeding stopped, and the skin was immediately sutured using 5‐0 nylon suture (Jinhuan Medical Products Co., Ltd. (Shanghai, China) with subsequent erythromycin ointment covered. After surgery, all animals were kept warm in a recovery cage. In the stress + incision (SI) group, surgery was performed the next day after stress exposure (Figure 1A). In contrast, the incision (I) group of mice underwent the same procedure without prior stress exposure.

### 4.4 OFT and Elevated Plus‐Maze Test

The OFT and EPM test were used to assess stress‐induced anxiety‐like behaviors [58, 59]. For OFT, the apparatus was made as a blue polyethylene box (50 × 50 × 40 cm) illuminated by a 25‐W halogen bulb. Each test mouse was gently placed in the box's central area, and its exploratory behavior was videotaped for 5 min. Between trials, the apparatus was cleaned with 75% ethanol to remove olfactory cues. The total time spent and the number of visits to the central area were analyzed using Clever TopScan software (Clever Sys, Reston, VA).

For EPM, the apparatus included two enclosed arms (30 cm each), two open arms (30 cm each), connected by a central platform (10 × 10 cm). All arms were positioned at a height of 100 cm above the ground. Testing began by gently placing each animal on the central area with its tail oriented toward the enclosed arms. Animals were allowed to explore freely for 5 min under continuous overhead monitoring, during which their entries into and duration in the open arms were recorded. Data were subsequently analyzed offline using Clever TopScan software (Clever Sys, Reston, VA). Between trials, the apparatus was cleaned with 75% ethanol to remove potential confounding odors.

### 4.5 Von Frey Tests

Individual mice were placed on a metallic grid (6 × 6 mm) beneath an inverted polyethylene cup (10 mm diameter × 12 mm height) and allowed to habituate to the environment for at least 30 min until they became calm. Mechanical sensitivity was assessed using a series of calibrated von Frey filaments designed to apply bending forces ranging from 0.07 to 2 g. Stimuli were applied vertically to the bottom (midplantar) surface of the hindpaw, with incremental pressure maintained for 1–3 s [60]. Each filament was tested five times with a 5‐min interval between trials, starting with the lowest force until a nociceptive response was observed.

### 4.6 Hot Plate Tests

Animals were first placed on a metallic plate maintained at 30°C within an acrylic cylinder (Xin Xin, Shanghai, China) to acclimate. Subsequently, animals were positioned on the same plate heated to 55°C, and the time to exhibit withdrawal, flinching, shaking, licking, or jumping was recorded [60]. A 20‐s cutoff was applied to prevent potential skin damage [61]. The final hindpaw withdrawal latency was calculated as the average of three independent measurements. To ensure reliability, each animal underwent three daily tests with a 5‐min rest interval between trials.

### 4.7 Amplitude of Low‐Frequency Fluctuations

Mice were acclimated to the testing environment for 3 consecutive days before fMRI scanning. The animals were anesthetized with isoflurane at 3% for induction and 1% for maintenance, delivered via 1 L/min O_2_. During the scanning session, body temperature and respiratory rate were continuously monitored and maintained at steady levels using heating pads and adjusting the depth of anesthesia, respectively. Animals were immobilized with MRI‐compatible ear and bite bars to ensure stable head positioning. Resting‐state fMRI was performed on a 9.4 T animal MRI scanner (Bruker BioSpec 94/20 USR, Germany) equipped with a specialized mouse head coil (26 mm in diameter). For spatial normalization of images, high‐resolution T2‐weighted images were obtained using a RARE sequence with the following parameters: repetition time (TR) = 4000 ms, echo time (TE) = 33 ms, FOV = 18 mm × 18 mm, average 4, matrix = 256 × 256, layers = 38, and slice thickness = 0.5 mm. For BOLD–fMRI acquisition, parameters included a TR = 1500 ms, TE = 7.88 ms, FOV = 30 mm × 12 mm, average 1, matrix = 125 × 50, layers = 35, and slice thickness = 0.5 mm. All the functional images were postprocessed by one experienced observer, who was blinded to the group assignment. The data preprocessing and analysis were carried out using the spmmouseIHEP [62] toolbox in the statistical parametric mapping (SPM12) software (Welcome Department of Imaging Science, London, UK; http://www.fil.ion.ucl.ac.uk/spm) [44], which included an fMRI template of the mouse brain and an atlas mapped in Paxinos & Watson space. Prior to fMRI analysis, preprocessing steps were performed to calculate the ALFF, including: (1) time‐slice correction; (2) small head movement correction; (3) spatial standardization; (4) Gaussian smoothing; (5) de‐linear drift; (6) using regression model to adjust for noise covariates; (7) band‐pass filtering at 0.01–0.2 Hz. Statistical thresholds were set at a voxel‐level height of *p* < 0.01 (uncorrected) and a cluster‐extent threshold of 20 voxels.

### 4.8 Seed‐Based FC

Based on the above ALFF analysis, we selected brain regions showing the most significant group differences in ALFF as seed regions (e.g., AIC) for further FC analysis [43]. For each animal, we calculated the time course of whole‐brain gray matter voxels for the selected seed region (AIC). The signal time courses were correlated with AIC time courses to generate a map of Pearson correlation coefficients (*r* values). These *r* values were then transformed into *z*‐scores using Fisher's *z*‐transformation to produce seed‐specific maps reflecting the intensity of associations with other brain regions.

### 4.9 In Vivo Calcium Signal Recording

As previously described [16], we recorded neuronal activity in the AIC using a 4‐mm optical fiber (ORIGINOPTO, Hangzhou, China) embedded in a dual‐color fiber photometry device (Inper‐C1‐2C, Hangzhou, China), which delivers excitation light at 410/470 nm to activate GCaMP6s. In Von Frey tests, awake mice were placed on a metallic grid situated beneath an inverted polyethylene cup equipped with drilled holes to facilitate passage of the optical fiber patch cord. Each stimulus was manually labeled with a unique time stamp and assigned simultaneously to ensure accurate temporal referencing. After the experiment, all animals were perfused for histological verification. Only data from animals with accurate recording sites were collected for further analysis with the Inper Data Process (Hangzhou, China). After removing background noise, we smoothed the activity traces with a 20‐ms moving‐average filter. Calcium flux values (Δ*F*/*F*) from −2 to 10 s (0 s, stimulus onset) were obtained by computing (*F* − *F*_0_)/*F*_0_ for each event, where *F*_0_ represented the mean fluorescence during the −2 s interval preceding the stimulus, and *F* was the calcium transients for the entire session.

### 4.10 Transfection and Intracranial Implantation

AIC injection sites were located at bregma: +2.00 mm, lateral: −2.25 mm, depth: −3.4 mm, and the OFC injection sites at bregma: +2.33 mm, lateral: −1.2 mm, depth: −2.65 mm, respectively. All viral vectors or drugs were delivered in a total volume of 200–400 nL per injection site.

To investigate the glutamatergic or GABAergic activity of AIC neurons in response to mechanical stimulation, AAV2/9–CaMKII*α*–GCaMP6s (titer: 2.0 × 10^12^ vg/mL; BrainVTA Biotechnology, Wuhan, China) or AAV2/9–VGAT–GCaMP6s (titer: 2.0 × 10^12^ vg/mL; BrainVTA Biotechnology) was injected into the right AIC, followed by implantation of optical fibers (ORIGINOPTO, Hangzhou, China) positioned 20 µm above the injection site. The fibers were secured to the skull using screws and dental cement [63]. For negative controls, AAV2/9–CaMKIIα–EGFP or AAV2/9–VGAT–EGFP (both titer: 2.0 × 10^1^ ^2^ vg/mL; Brain Case, Shenzhen, China) was used.

To selectively label the AIC glutamatergic neurons for c‐Fos expression analysis, a mixture of AAV2/9‐CaMKII*α*‐Cre virus (titer: 2.0 × 10^12^ vg/mL; Brain Case) and AAV2/9–EF1*α*–DIO–mCherry virus (titer: 2.0 × 10^12^ vg/mL; Brain Case) was administered into the right AIC 21 days prior to histological staining.

For manipulating the glutamatergic activity of AIC neurons with designer receptors exclusively activated by designer drugs (DREADD), a mixture of AAV2/9–CaMKII*α*–Cre virus (titer: 2.0 × 10^12^ vg/mL; Brain Case) and AAV2/9–EF1*α*–DIO–hM3Dq–mCherry virus (titer: 2.0 × 10^12^ vg/mL; Brain Case) or AAV2/9–EF1*α*–DIO–hM4Di–mCherry virus (titer: 2.0 × 10^12^ vg/mL; Brain Case) was injected into the right AIC 21 days before pain behavior tests. AAV2/9–EF1*α*–DIO–mCherry virus (titer: 2.0 × 10^12^ vg/mL; Brain Case) was used as a control.

For activating the AIC GABAergic neuron activities with DREADD, a mixture of AAV2/9–VGAT–Cre (titer: 2.0 × 10^12^ vg/mL; BrainVTA) and AAV2/9–EF1*α*–DIO–hM3Dq–mCherry virus (titer: 2.0 × 10^12^ vg/mL; Brain Case) was injected into the right AIC 21 days before pain behavior tests. AAV2/9–EF1*α*–DIO–mCherry virus (titer: 2.0 × 10^12^ vg/mL; Brain Case) virus was used as a control.

For anterograde monosynaptic tracing of the OFC–AIC^Glu^ pathway, 21 days after viral transfection into the OFC of *Td‐tomato reporter mice* with a mixture of AAV2/1–CaMKII*α*–Cre virus (titer: 3.0 × 10^12^ vg/mL; BrainVTA) and AAV2/9–CaMKII*α*–mCherry virus (titer: 2.0 × 10^12^ vg/mL; BrainVTA), immunofluorescence staining targeting glutamate was then performed. The AAV2/9–CaMKII*α*–mCherry virus was used to visualize the injection site. For retrograde tracing of the OFC^Glu^–AIC pathway, 21 days after viral transfection into the AIC of *wild‐type mice* with AAV2/R–CaMKII*α*–mCherry virus (titer: 2.0 × 10^12^ vg/mL; BrainVTA), immunofluorescence staining targeting glutamate was then performed.

For optogenetic activation of the OFC–AIC pathway, 28 days after viral transfection into the OFC with a mixture of AAV2/9–CaMKII*α*–Cre virus (titer: 2.0 × 10^12^ vg/mL; Brain Case) and AAV2/9–EF1*α*–DIO–ChR2–mCherry virus (titer: 2.0 × 10^12^ vg/mL; Brain Case), electrophysiological recordings with blue light stimulation were performed. AAV2/9–EF1*α*–DIO–mCherry virus (titer: 2.0 × 10^12^ vg/mL; Brain Case) was used as a control.

For chemogenetic activation of the OFC–AIC pathway, 17 days after viral transfection into the OFC with a mixture of AAV2/9–CaMKII*α*–Cre virus (titer: 2.0 × 10^12^ vg/mL; Brain Case) and AAV2/9–EF1*α*–DIO–hM3Dq–mCherry virus (titer: 2.0 × 10^12^ vg/mL; Brain Case), a cannula (0.40 mm in outer diameter) was implanted above the right AIC (bregma: +2.00 mm, lateral: −2.25 mm, depth: −3.4 mm), and attached to the skull using screws and dental cement. Pain behavior tests were then performed 4 days after cannula implant surgeries. AAV2/9–EF1*α*–DIO–mCherry virus (titer: 2.0 × 10^12^ vg/mL; Brain Case) was used as a control.

### 4.11 Drug Administration

Experiments involving CNO (6329; Tocris Bioscience) administration were performed via intra‐AIC or intraperitoneal injections in mice, depending on the experimental requirements. For intra‐AIC administration, CNO was dissolved in 5% DMSO (1.7 µg/mL) and injected at 300 nL. For intraperitoneal administration, CNO was delivered at a dose of 5 mg/kg in 0.5 mL 5% DMSO. An identical volume of 5% DMSO was served as a control.

To assess the systemic effects of Dex in response to non‐nociceptive (mechanical) or nociceptive (thermal) stimuli, Dex (1.25 µg/mL in saline, 231123BP; Hengrui Medicine Co., Ltd, Jiangsu, China), was intraperitoneally delivered to a volume of 0.5 mL (25 µg/kg). To examine the local effects of Dex in response to non‐nociceptive (mechanical) or nociceptive (thermal) stimuli, guided cannula (0.40 mm in diameter) was implanted 0.2 mm above the right AIC (bregma: +2.00 mm, lateral: −2.25 mm, depth: −3.4 mm), through which Dex, dissolved in saline (12 µM), was delivered to a volume of 500 nL at a rate of 100 nL/min. Both systemic and local dosing regimens were selected based on previously reported effective concentrations [21, 64, 65], and an equivalent volume of saline served as a control.

To evaluate whether Dex's plasticity‐modulating effect on the OFC–AIC circuit depends on *α*2‐AR activation, Dex (12 µM) was locally microinfused into the AIC 6 h prior to behavioral testing, in the presence or absence of the *α*2‐AR antagonist yohimbine (6 µM; GN10606; GlpBio Technology, Jiangsu, China). Subsequent pain‐sensitivity measurements allowed assessment of the contribution of *α*2‐AR signaling to Dex's sustained antihyperalgesic action.

### 4.12 Immunofluorescent Staining

Mice were lethally anesthetized with sodium pentobarbital (100 mg/kg), and transcardially perfused with ice‐cold saline followed by 4% phosphate‐buffered paraformaldehyde (pH 7.4). The brain was collected at the end of the perfusion and then postfixed in 4% phosphate‐buffered paraformaldehyde overnight at 4°C, followed by dehydration in 30% sucrose. Frozen sections (30 µm) were prepared with the cryostat (FS800/FS800A; RWD), and immediately mounted on slides for immunohistochemical staining. Cryosections were blocked and permeabilized in 5% BSA (0.3% Triton X‐100 in PBS) for 1 h at room temperature (25°C), followed by overnight incubation with primary antibodies at 4°C and secondary antibodies at 25°C for 1.5 h. Primary antibodies used in this section were listed as follows: rabbit anti‐c‐Fos (1:500, 9F6; CST), mouse antiglutamate (1:200, MAB5304; Millipore), mouse anti‐GABA (1:200, A0310; Sigma–Aldrich), and rabbit antiadrenergic receptor alpha‐2A (1:200, DF3076; Affinity). Secondary antibodies used here included donkey antirabbit Alexa fluor 488 (1:1000, A‐21206; Invitrogen), donkey antimouse Alexa fluor 546 (1:1000, A10036; Invitrogen).

### 4.13 Imaging and Analysis

Image capture was performed using either an Olympus VS‐120 microscope or an Olympus FV980 confocal microscope. Cells expressing c‐Fos, glutamate, GABA, mCherry, or EGFP were counted manually under blinded conditions. Similar areas were randomly selected from brain regions of interest in six mice and averaged. To plot the distribution pattern of c‐Fos expressing neurons, similar fields from five consecutive slices (Bregma 2.33 to 1.93 mm) in six mice were analyzed, and the number of c‐Fos per slice was calculated as the mean value; the location of c‐Fos was manually marked on a common brain atlas.

### 4.14 In Vitro Electrophysiological Recordings

The electrophysiology recording protocol followed our previous reports [43, 58, 66, 67, 68]. After being deeply anesthetized with isoflurane (R510‐22‐4; RWD), mice were perfused with 10 mL ice‐cold oxygenated cutting solution (containing 110 mM choline chloride, 2.5 mM KCl, 1.3 mM NaH_2_PO_4_, 22 mM glucose, 26 mM NaHCO_3_, 0.5 mM CaCl_2_, 7 mM MgCl_2_, 5 mM sodium ascorbate, and 3 mM sodium pyruvate). To isolate the plasticity‑specific effects of Dex on AIC neurons, brains were rapidly dissected 6 h after Dex administration—a time point when its acute analgesic action had subsided. Subsequently, 300‑µm coronal slices were prepared in ice‑cold cutting solution saturated with 95% O_2_ and 5% CO_2_, using a vibratome (VT1000S; Leica, Germany). Slices were next incubated with oxygenated artificial cerebral spinal fluid (ACSF) (containing 119 mM NaCl, 2.5 mM KCl, 1 mM NaH_2_PO_4_, 2.7 mM MgCl_2_, 26 mM NaHCO_3_, and 11 mM glucose, 3.3 mM CaCl_2_ at 35°C for 30 min), then equilibrated at room temperature for 30 min before recording. Whole‐cell recordings were performed on pyramidal neurons expressing mCherry in AIC under a fluorescence microscope (BX51WI; Olympus, Japan) with borosilicate glass pipettes (3–5 MΩ).

All recordings were conducted using an Axopatch‐700B amplifier (Axon Instruments, Foster City, CA, USA). The recorded signals were transmitted to a computer via a Digidata‐1550 interface (Axon Instruments) for subsequent data acquisition and analysis, which was performed using pClamp 10.0 software (Axon Instruments). Postsynaptic currents were digitally sampled at 10 kHz and then low‐pass filtered at 1 kHz. Membrane potential recordings were sampled at 20 kHz and then low‐pass filtered at 5 kHz. Neurons were injected with rectangular voltage pulses (5 mV, 50 ms) at a holding potential of −70 mV to measure whole‐cell membrane capacitance, membrane resistance, and series resistance. Neurons were excluded from analysis if series resistance exhibited fluctuations exceeding 20% of the initial values.

For voltage‐clamp recordings, Cs‐based internal solution (containing 120 mM Cs^+^‐gluconate, 20 mM CsCl, 10 mM HEPES, 0.2 mM EGTA, 10 mM disodium phosphocreatine, 5 mM QX314, 4 mM Na_2_‐ATP, 0.4 mM GTP–Tris, adjusted to pH 7.2 with CsOH) was used. sEPSCs were recorded at −70 mV in the presence of the selective GABA_A_ receptor antagonist picrotoxin (100 µM, 1128; Tocris Bioscience), while spontaneous sIPSCs were recorded at 0 mV in the presence of the selective AMPA receptor antagonist NBQX (20 µM, 1044; Tocris Bioscience) and the selective N‐methyl‐d‐aspartate receptor antagonist d‐APV (50 µM, 0106; Tocris Bioscience).

For current‐clamp recordings, K‐based internal solution (containing 140 mM K‐gluconate, 7 mM KCl, 2 mM MgCl_2_, 10 mM HEPES, 0.1 mM EGTA, 4 mM Na_2_‐ATP, 0.4 mM GTP–Tris, adjusted to pH 7.2 with KOH) was used to record AP firing rate. Evoked APs were detected in response to stepwise depolarizing currents (0–300 pA, 200 ms duration, incrementally applied in 50 pA steps) or linearly increasing depolarizing currents (0–300 pA, 5000 ms duration, increasing at 0.5 pA/ms for the first 600 ms). Biocytin (0.05%; Sigma) was added to the internal solution for further morphological identification of the recorded pyramidal neurons after recordings.

For light‐evoked EPSC recordings [43, 58], ChR2 was activated using 10 ms 470 nm light pulses delivered via an LED (pE‐300white; CoolLED, Andover, UK), mounted on the upright objective, with light intensity <2 mW. To evoke efficient synaptic responses via ChR2 activation, postsynaptic recordings were performed from pyramidal neurons in areas of high ChR2 axonal infectivity in AIC.

To demonstrate the monosynaptic glutamatergic projections from the OFC to the AIC, we employed NBQX (20 µM) as a selective AMPA receptor antagonist to confirm OFC glutamatergic projections onto AIC pyramidal cells. TTX (0.5 µM, T‐500; Alomone Laboratory), a specific voltage‐gated sodium channel blocker, and 4‐AP (300 µM, A1910; Sigma), a relatively specific potassium channel blocker, were applied to identify the monosynaptic connections between OFC and AIC.

To examine whether Dex's effects on the circuit activation between OFC and AIC is *α*2‐AR activation dependent, Dex (12 µM) was infused to assess the alteration of light‐evoked EPSCs in the presence or absence of yohimbine (6 µM), the antagonist of *α*2‐ARs.

### 4.15 Statistical Analysis

Statistical analyses were performed using Prism 8 (GraphPad Software, Inc., San Diego, CA, USA). Data are presented as mean ± SEM and analyzed with either two‐tailed unpaired or paired *t‐*tests, and one‐way or two‐way analysis of variance (ANOVA). Multiple comparisons between groups were compared using either Tukey's posthoc testing for one‐way ANOVA, or using Bonferroni's correction for two‐way ANOVA. *p* Values less than 0.05 were considered statistically significant.

## Author Contributions

Zhengliang Ma, Jing‐Ning Zhu, Xiao‐Yang Zhang, and Xiaoping Gu conceptualized the study. Hui Rong, Yang‐Xun Zhang, Huijie Zhu, and Yinyao Li carried out all experiments, performed statistical analyses, and prepared all figures with contributions from Yunfan Hou, Yong‐Yun Xie, Lu‐Yao Li, and Bei‐Bei Zhang. Shu‐Tao Xie, Qi‐Peng Zhang, and Wei Zhang provided constructive comments. Hui Rong wrote the original draft. Xiao‐Yang Zhang, Xiaoping Gu, Jing‐Ning Zhu, and Zhengliang Ma revised the manuscript. Jing‐Ning Zhu and Zhengliang Ma performed supervision. All listed authors have agreed to the final submitted version.

## Ethics Statement

All the conducted experiments in the present study were approved by the Care Committee of Affiliated Drum Tower Hospital, Medical School of Nanjing University (license No. 2020AE01110).

## Conflicts of Interest

The authors declare no conflicts of interest.

## Supporting information




**Supporting Information**: mco270848‐sup‐0001‐SuppMat.docx

## Data Availability

Source data are provided with this paper. The data relevant to present study are available on reasonable request to the corresponding authors.
