# Supplementary material for: Dexmedetomidine Rapidly Relieves Stress‐Induced Hyperalgesia via Presynaptic α2‐Adrenergic Inhibition at Orbitofrontal–Insular Glutamatergic Synapses
Source: MedComm (2020). 2026 Jun 30;7(7):e70848. doi: 10.1002/mco2.70848 (PMC13319410; doi:10.1002/mco2.70848)
Supplement: Supplementary file 1 — Supporting Information: mco270848‐sup‐0001‐SuppMat.docx [file MCO2-7-e70848-s001.docx]

Dexmedetomidine rapidly relieves stress-induced hyperalgesia via presynaptic *α*2-adrenergic inhibition at orbitofrontal-insular glutamatergic synapses

Hui Rong^1†^, Yang-Xun Zhang^2†^, Huijie Zhu^1†^, Yinyao Li^1†^, Yunfan Hou^1^, Yun-Yong Xie ^2^, Lu-Yao Li^2^, Bei-Bei Zhang^2^, Shu-Tao Xie^2^, Wei Zhang^1^, Qi-Peng Zhang^2^,

Xiao-Yang Zhang^2^*, Jing-Ning Zhu^2^*, Xiaoping Gu^1^*, Zhengliang Ma^1^*

^1^ Department of Anesthesiology, Nanjing Drum Tower Hospital, Affiliated Hospital of Medical School, Nanjing University, Nanjing, China

^2^ State Key Laboratory of Pharmaceutical Biotechnology, National Resource Center for Mutant Mice, Department of Anesthesiology, Nanjing Drum Tower Hospital, Institute for Brain Sciences, and Department of Physiology, School of Life Sciences, Nanjing University, Nanjing, China

†Contributed equally.

***Correspondence to:** mazhengliang1964@nju.edu.cn (Zhengliang Ma). xgu1180@vip.163.com (Xiaoping Gu). jnzhu@nju.edu.cn (Jing-Ning Zhu). xiaoyangzhang@nju.edu.cn (Xiao-Yang Zhang). Tel.: +86-025-89684720; Fax: +86-025-89684720.

Running title: Dex relieves hyperalgesia via OFC–AIC circuit

**

**

**Figure S1** Verification of SPS effectiveness by anxiety-like phenotypes and insufficient fentanyl analgesia in SI-treated mice. (A) Representative motion tracks in the open field test (OFT) for control (Ctrl) and single persistent stress (SPS) mice. (B) Time spent in the central area in Ctrl (*n* = 16) and SPS (*n* = 17) mice. (C) Numbers of entries into the central area in Ctrl (*n* = 16) and SPS (*n* = 17) mice. (D) Total distance traveled in Ctrl (*n* = 16) and SPS (*n* = 17) mice. (E) Representative motion tracks in the elevated plus maze (EPM) for Ctrl and SPS mice. (F) Time spent in the open arm in Ctrl (*n* = 17) and SPS (*n* = 18) mice. (G) Number of entries into the open arm in Ctrl (*n* = 17) and SPS (*n* = 18) mice. (H) Total distance traveled in Ctrl (*n* = 17) and SPS (*n* = 18) mice. (I) Determination of the minimum effective dose of fentanyl (3.12–50 *μ*g/kg in 0.5 mL saline, i.p.) in incisional mice. (J) Mechanical hyperalgesia in the ipsilateral hindpaw after stress exposure, shown by paw withdrawal mechanical threshold (PWMT) in stress + incision (SI, day 1) mice 2 hours after saline or fentanyl (12.5 *μ*g/kg, i.p.) treatment. Data are shown as mean ± SEM. NS, no significant. *****p* < 0.0001. Statistical tests: two-tailed unpaired *t*-test (B–D, F–H, and J) and one-way ANOVA with Bonferroni’s post hoc test (I). See also Data file.





**Figure S2** Increased ALFF in the right AIC in SIH mice. (A) Representative resting-state fMRI images showing amplitude of low-frequency fluctuations (ALFF) in stress + incision (SI) and incision (I) mice. (B) Quantification of ALFF changes across anxiety-, or negative affect, and pain-related brain regions. *n* = 9 mice per group. SI was used to induce stress-induced hyperalgesia (SIH). Data from AIC are also shown in Figure 2A, B. I, incision; SI, stress + incision; ACC, anterior cingulate cortex; BNST, bed nucleus of the stria terminalis; DRN, dorsal raphe nuclei; HPC, hippocampus; HYP, hypothalamus; MD, mediodorsal thalamic nucleus; OFC, orbitofrontal cortex; PAG, periaqueductal grey; PBN, parabrachial nucleus; PFC, prefrontal cortex; SC, somatosensory cortex; TH, thalamus; VTA, ventral tegmental area; RVM, rostral ventromedial medulla; ZI, zona incerta. Data are shown as mean ± SEM. **p* < 0.05, ***p* < 0.01. Statistical test: two-tailed unpaired *t*-test (B). See also Data file.





**Figure S3** Dex suppresses stress-induced increases in spontaneous activity of AIC^Glu^ neurons in response to exogenous stimuli. (A) Experimental schematic for panels (B–N). I, incision; SI, stress + incision; Dex, dexmedetomidine. (B) Diagram of whole-cell recordings in pyramidal neurons of the anterior insular cortex (AIC). (C) Representative traces showing different phases of spontaneous action potential (AP) firing in AIC pyramidal neurons. (D) Corresponding phase-plane plots from each experimental group. (E) Membrane capacitance (Cm) before and after Dex application (*n* = 18–21 neurons from 4–7 mice per group). (F) Membrane resistance (Rm) before and after Dex application (*n* = 24–28 neurons from 4–7 mice per group). (G) Resting membrane potential (RMP) before and after Dex application (*n* = 22–30 neurons from 3–4 mice per group). (H) Firing threshold before and after Dex application (*n* = 4–12 neurons from 4–6 mice per group). (I) Difference between RMP and threshold before and after Dex application (*n* = 4–6 neurons from 4–6 mice per group). (J) AP amplitude before and after Dex application (*n* = 4–12 neurons from 4–6 mice per group). (K) Half duration before and after Dex application (*n* = 4–12 neurons from 4–6 mice per group). (L) Rise time before and after Dex application (*n* = 4–12 neurons from 4–6 mice per group). (M) Maximum rising slope before and after Dex application (*n* = 4–12 neurons from 4–6 mice per group). (N) Maximum decaying slope before and after Dex application (*n* = 4–12 neurons from 4–6 mice per group). Data are shown as mean ± SEM. NS, no significant. **p* < 0.05, ***p* < 0.01, ****p* < 0.001. One-way ANOVA with Tukey’s post hoc test (E–N). See also Data file.





**Figure S4** Local Dex treatment rescues stress-induced alterations in intrinsic excitability of AIC^Glu^ neurons. (A) Experimental schematic for panels (B–F). I, incision; SI, stress + incision; Dex, dexmedetomidine. (B) Representative current-clamp traces and firing rate plots of APs evoked by a linear ramp current (0 to +300 pA). (C) Quantification of Dex effects on stress-induced changes in ramp gain in AIC pyramidal neurons of SI mice (*n* = 17–25 neurons from 4 mice per group). (D) Quantification of Dex effects on stress-induced changes in ramp latency in AIC pyramidal neurons of SI mice (*n* = 17–26 neurons from 3–4 mice per group). (E) Quantification of Dex effects on stress-induced changes in ramp adaptation in AIC pyramidal neurons of SI mice (*n* = 11–16 neurons from 3–7 mice per group). (F) Quantification of Dex effects on stress-induced changes in ramp adaptation ratio in AIC pyramidal neurons of SI mice (*n* = 10–15 neurons from 3–7 mice per group). Data are shown as mean ± SEM. NS, no significant. **p* < 0.05, ***p* < 0.01, ****p* < 0.001. One-way ANOVA with Tukey’s post hoc test (C–F). See also Data file.

**

**

**Figure** **S5** Spontaneous activity of AIC^GABA^ neurons and sensitivity to exogenous stimuli are not altered after stress or Dex application. (A) Experimental schematic for panels (B–K). (B) Morphology of biocytin-labeled patched non-pyramidal neurons. Scale bar: 20 *μ*m. (C) Example current-clamp traces of APs evoked by step current injections (0 to +500 pA). (D) Quantification of AP firing rate (*n* = 20 cells from 5 mice per group). (E) Quantification of AP step gain (*n* = 20 cells from 5 mice per group). (F) Quantification of AP rheobase (*n* = 20 cells from 5 mice per group). (G) Schematic of fiber photometry recording with a 0.4-g subthreshold stimulation in mice (*n* = 5 mice). (H) Representative images of fiber photometry recording in mice. Scale bars: 100 *μ*m (left) and 50 *μ*m (right) (*n* = 5 mice). (I) Mean ΔF/F (%) of AIC^GABA^ neuronal activity in response to 0.4-g mechanical stimulation. (J) Quantification of calcium peak values in (I) (*n* = 11–21 trials from 5 mice). Data are shown as mean ± SEM. NS, no significant. Statistical tests: two-way ANOVA with Bonferroni’s post hoc test (D) and one-way ANOVA with Tukey’s post hoc test (E,F, and J). See also data file.

**

**

**Figure S6** Enhanced functional connectivity of the right AIC following stress. (A) Representative resting-state fMRI images showing seed-based functional connectivity (FC) analysis from the right anterior insular cortex (AIC). (B) Quantification of FC strength showing significant increases between the right orbitofrontal cortex (OFC) and right AIC. *n* = 9 mice per group. I, incision. SI, stress + incision. Data for OFC–AIC connectivity are also shown in Figure 6B. OT, olfactory tubercle; OB, olfactory bulb; ACC, anterior cingulate cortex; PrL, prelimbic cortex; PBN, parabrachial nucleus; HPC, hippocampus; AMY, amygdala; HYP, hypothalamus; S_1,2_, primary and secondary somatosensory cortex; M, motor cortex; LC, locus coeruleus. Data are shown as mean ± SEM. **p* < 0.05, ***p* < 0.01. Two-tailed unpaired *t*-test (B). See also Data file.





**Figure S7** Dex-mediated presynaptic modulation may involve suppression of excitatory transmission within OFC^Glu^–AIC circuit. (A) Schematic of anterograde monosynaptic tracing and representative images showing AAV2/1-CaMKII*α*-Cre and AAV2/9-CaMKII*α*-mCherry injections into the orbitofrontal cortex (OFC), including ventral orbital (VO) and lateral orbital (LO) subdivisions, in *Td-tomato reporter mice*. Mixed AAV2/9-CaMKII*α*-mCherry was used to verify infusion sites in the OFC. Anterogradely labeled Td-tomato^+^ neurons were observed in the anterior insular cortex (AIC). Representative images show Td-tomato^+^ neurons co-labeled with glutamate in the right-lateral AIC. Scale bars: 500 *µ*m (middle) and 50 *µ*m (right). (B) Schematic of retrograde tracing and representative images showing AAV2/R-CaMKII*α*-mCherry injections into the AIC, including anterior agranular insular (AID) and anterior ventral insular (AIV) subdivisions, in *wild-type mice*. Retrogradely labeled mCherry^+^ neurons were identified in the VO and LO of the OFC, with magnified views in the right panel. Scale bars: 500 *µ*m (middle) and 100 *µ*m (right). (C) Representative images showing mCherry^+^ neurons (red) co-localized with glutamate (green) in the right-lateral OFC. Co-labeled neurons are indicated by white arrows. Scale bars: 50 *µ*m. See also Data file.





**Figure S8** Functional identification of monosynaptic connection from OFC^Glu^ to AIC pyramidal neurons. (A) Schematic showing AAV infusion into the orbitofrontal cortex (OFC), local drug delivery, and patch clamp recordings in *ex vivo* brain slices containing the anterior insula cortex (AIC). TTX, Tetrodotoxin; 4-AP, 4-aminopyridine. (B) Schematic of optogenetic activation of the OFC–AIC glutamatergic circuit and local expression of channelrhodopsin 2 (ChR2)-mCherry in the OFC. mCherry^+^ somata were fully co-labeled with glutamate, and mCherry^+^ terminals were observed in the AIC. (C) Representative traces (C1) and quantification of light-evoked excitatory postsynaptic currents (EPSCs; C2) and latency (C3) demonstrating a direct OFC–AIC glutamatergic projection following NBQX (20 *μ*M) infusion (*n* = 9 cells from 5 mice). (D) Representative traces and quantification of monosynaptic connections from OFC^Glu^ to AIC pyramidal neurons (*n* = 8 cells from 4 mice) with sequential infusion of TTX (0.5 *μ*M) and 4-AP (300 *μ*M). Recordings were made from AIC pyramidal neurons. Left scale bar: 20 pA, 50 ms; right scale bar: 50 pA, 50 ms. Data are shown as mean ± SEM. *****p* < 0.0001. Statistical tests: two-tailed paired *t*-test (C2) and one-way ANOVA with Bonferroni's post hoc test (D). See also Data file.





**Figure S9** Effect of selective *α*2‑AR blockade on the pain threshold of Group SIH mice locally treated with Dex in AIC. (A) Schematic showing stress-induced hyperalgesia (SIH) induction, local drug delivery in the anterior insula cortex (AIC), and pain test at 6 hours after administration. I, incision only. SI, stress + incision. Dex, dexmedetomidine; Yo, yohimbine. (B) Changes of paw withdraw mechanical threshold (PWMT) in the contralateral hindpaws in SIH (day 1) mice 6 hours after Dex (12 *μ*M) or Dex + Yo (6 *μ*M) treatment. *n* = 10 mice per group. (C) Changes of thermal withdrawal latency in SIH mice. *n* = 10 mice per group. Data are shown as mean ± SEM. NS, no significant. *****p* < 0.0001. Statistical tests: two-tailed paired *t*-test. See also Data file.
